# 2-*O*-Monoalkyl isosorbide ethers with C8, C10, C12 and C14 chain lengths

**DOI:** 10.1107/S2056989020006647

**Published:** 2020-05-29

**Authors:** Felix Geburtig, Volkmar Vill

**Affiliations:** a Universität Hamburg, Martin-Luther-King-Platz 6, 20146 Hamburg, Germany

**Keywords:** crystal structure, isorbide, short-chain amphiphiles, carbohydrate derivate

## Abstract

The title compounds, 6-(octyloxy)hexa­hydro­furo[3,2-*b*]furan-3-ol, C_14_H_26_O_4_, 6-(decyl­oxy)hexa­hydro­furo[3,2-*b*]furan-3-ol, C_16_H_30_O_4_, 6-(do­decyl­oxy)hexa­hydro­furo[3,2-*b*]furan-3-ol, C_18_H_34_O_4_, and 6-(tetra­decyl­oxy)hexa­hydro­furo[3,2-*b*]furan-3-ol, C_20_H_38_O_4_, consist of a polar headgroup (isosorbide) and a lipophilic alkyl chain linked *via* an ether bridge. Isosorbide is a biobased diol, containing two fused furan rings. One inter­molecular hydrogen bond connects the mol­ecules between the free *endo* hy­droxy group and the opposing ether oxygen of the V-shaped head group. Thus the mol­ecule layers inter­lock like in a herringbone pattern parallel to the *bc* plane.

## Abstract 2

The crystal structures of 2-*O*-monooctyl isosorbide, 2-*O*-monodecyl isosorbide, 2-*O*-monododecyl isosorbide and 2-*O*-mono­tetra­decyl isosorbide are reported. All four compounds crystallize in the chiral space group *P*2_1_.

## Chemical context   

We are inter­ested in the synthesis and characterization of amphiphiles and liquid crystals based on renewable resources with a special focus on glycolipid structures. The mol­ecules of the reported crystal structures are precursor compounds to possible liquid crystals, which may already present some liquid crystal properties. The exact geometric shape of the mol­ecule under consideration is decisive for the explanation of observed desired liquid crystal properties. (Vill *et al.*, 1988 ▸; Vill *et al.*, 1989 ▸; Etzbach *et al.*, 1995 ▸). These reported precursors and their corresponding endo-isomers (5-*O*-alkyl­isosorbide) were also examined for thermotropic and lyotropic liquid crystal properties. In contrast to the exo-isomers presented here, the endo-isomers are colorless fluids at standard conditions for temperature and pressure. The exo-isomers crystallize in colorless needles at given conditions.
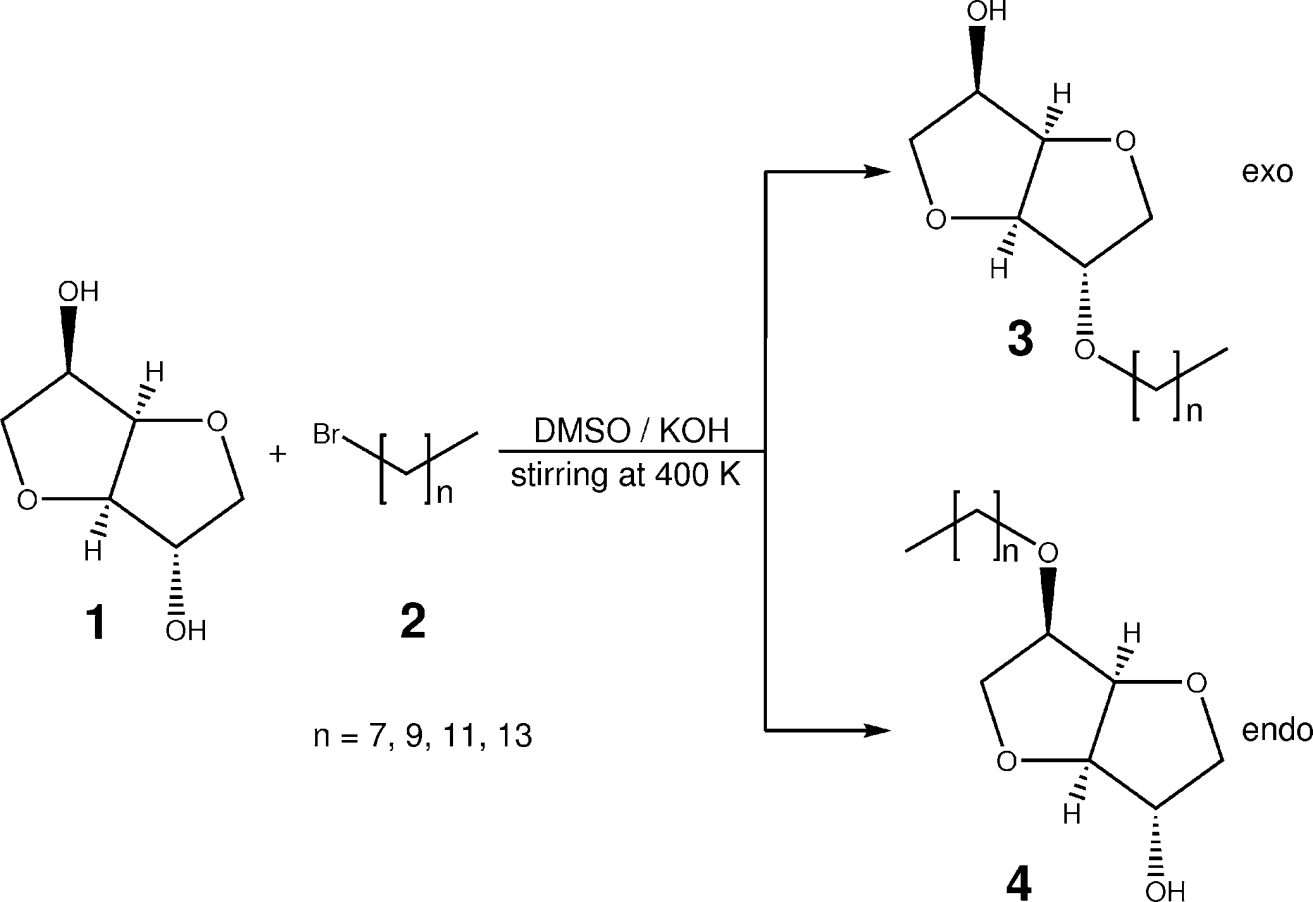


## Structural commentary   

**Figure 1:**
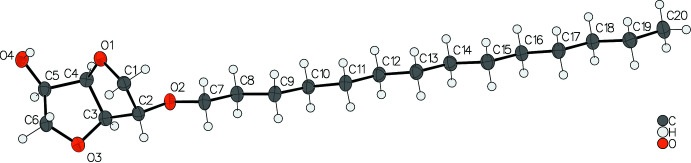
Molecular structure of the title compound **3d** with chain length C_14_ in the crystal. Ellipsoids represent 50% probability levels.

The Flack parameters and associated e.s.d. values in the title compounds are −0.7 (5) (**3a**), −0.18 (13) (**3b**), 1.6 (9) (**3c**) and −1.1 (10) (**3d**). None of the esd values meets the criterion for enanti­opure-sufficient inversion-distinguishing power (Flack & Bernardinelli, 2000 ▸), which is expected given that compounds **3a**, **3b** and **3d** were measured using Mo radiation and Friedif values are in the range of 6 to 7 (Mo) and 33 to 35 (Cu), respectively (Flack *et al.*, 2007 ▸; Flack, 2008 ▸). Absolute configurations were thus established from unchanging chiral centers of enanti­opure starting materials (*_chemical_absolute_configuration syn*). The Flack parameter of the reported compounds is essentially inconclusive. Nevertheless, the structure analysis confirms the formation of compound **3a**–**d**. Fig. 1 ▸ shows compound **3d** with a chain length of C_14_. The other compounds with chain lengths of C_8_, C_10_ and C_12_ have a strong structural similarity and are not shown explicitly. Geometric parameters are given in Table 1 ▸.

## Supra­molecular features   

**Figure 2:**
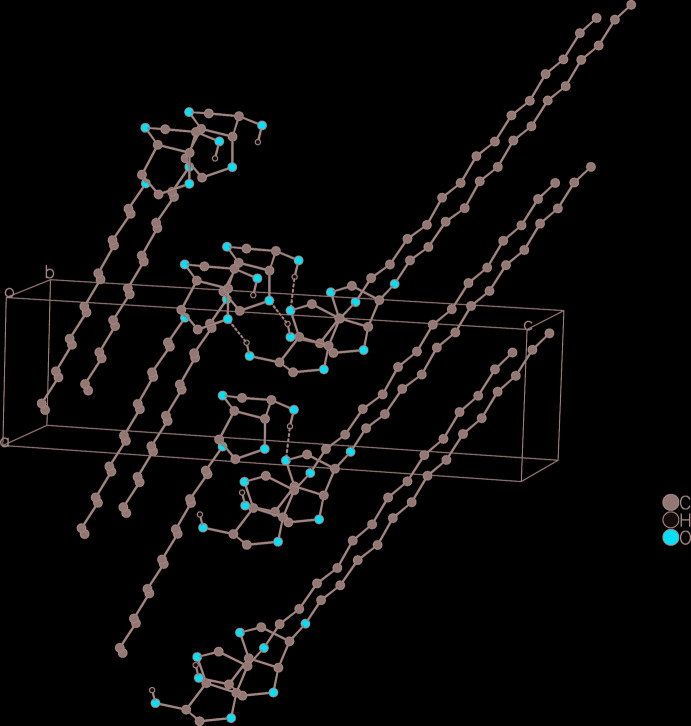
Crystal structure of the title compound **3d** with chain length C_14_ in the crystal. Ellipsoids represent 50% probability levels.

**Figure 3:**
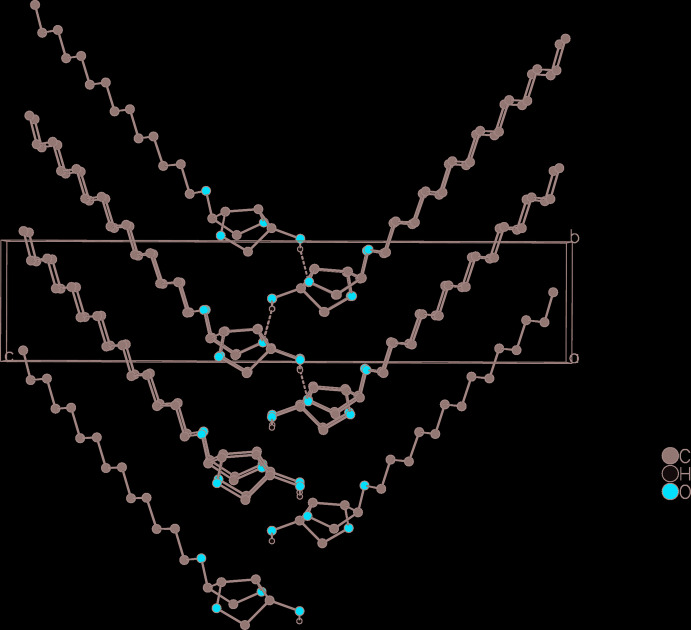
Packing diagram of **3d** projected parallel to the *ac* plane. Dashed lines indicate the inter­molecular hydrogen bonds. Hydrogen atoms not involved in the hydrogen-bonding system are omitted.

Van der Waals forces cause the mol­ecules to stack in layers. A classical inter­molecular hydrogen bond is observed (Table 2 ▸, Fig. 2 ▸) between the polar headgroups of two neighboring layers. Because each polar headgroup functions as hydrogen-bond acceptor and donor, the hydrogen bond reinforces the connection between the layers and strengthens the coherence within the layer, inter­locking the mol­ecules into a herringbone pattern parallel to the *bc* plane. The inter­molecular torsion angle O2—C2⋯C2—O2^i^ (Table 1 ▸) is between 52 and 55°. This intermolecular torsion angle directly corresponds to the opening angle of the herringbone pattern (Figs. 3 ▸ and 4 ▸).

Regarding the angle of the inter­molecular hydrogen bond O4—H4⋯O1^i^, it can be seen that the angle varies slightly with the chain length of the non-polar chain between 155 and 162°; the distance between the donor and acceptor of the hydrogen bond also stays roughly the same: 2.823–2.834 Å (Table 2 ▸).

## Database survey   

A search of the Cambridge Structural Database (CSD, version 5.41, update of November 2019; Groom *et al.*, 2016 ▸) for isosorbide derivates gave only seven hits, whereby only three hits were mono-substituted: NOZVUW (Sagawa *et al.*, 2019 ▸) is the 2-acetamide-2-de­oxy­isosorbide, PIMKOO (Kanters *et al.*, 1993 ▸) is the isosorbide-2-mononitrate and TUQGET (Santschi *et al.*, 2015 ▸) the isosorbide-5-mononitrate. Therefore, none of them represent mono-alkyl ethers. PIMKOL (Kanters *et al.*, 1993 ▸) is the isosorbide dinitrate of the corresponding isosorbide-2-mononitrate whereas WECBUE (Wu *et al.*, 2017 ▸) is the di­nitrile. MOVFUY (Harata & Kawano, 2002 ▸) is a bis­(α-cyclo­dextrin) clathrate of isosorbide dinitrate and TECRIC (Hušák *et al.*, 1996 ▸) is a cyclo­sporine di­methyl­isosorbide solvate. Only the latter is an alkyl ether, but disubstituted. Regarding the angle C2—C3—O3 constituted by the annulated tetra­hydro­furan rings, it is noticeable that the angles of the reported compounds here are larger than those of the mono- and dinitrates whereas the angle O1—C4—C5 is smaller. The cyclo­sporine di­methyl­isosorbide solvate has a larger angle for O1—C4—C5 whereas the angle for C2—C3—O3 is smaller in comparison to compounds presented here. The di­nitrile isosorbide shows a comparable angle for the angle O1—C4—C5, but the C2—C3—O3 angle is larger in that compound compared to the mono-alkyl ethers. The same applies to the isosorbide dinitrate clathrate. The 2-acetamide-2-de­oxy­isosorbide shows angles that are comparable to the mono-alkyl ethers reported here.

## Synthesis and crystallization   

Isosorbide **1** (30 mmol) and potassium hydroxide (30 mmol) were dissolved under stirring in 15 mL dimethyl sulfoxide at 400 K. Bromo alkane **2** (20 mmol) was added slowly. The solution was kept at 400 K under stirring for 24h. The solution was cooled to room temperature and acidified to pH = 1 with 37% hydro­chloric acid. Triple extraction with 50 mL of ethyl acetate and drying the collected organic phases over magnesium sulfate gave a golden-yellow raw product after removal of the solvent under reduced pressure. The raw product was separated and purified by column chromatography (solvent: petroleum ether 50–70/ethyl acetate 1:1). Evaporation of the solvent under reduced pressure afforded compound **3** as colorless crystals and compound **4** as colorless syrup-like fluids in a combined yield of 30 to 50% (Zhu *et al.*, 2008 ▸).

**2-** ***O*** **-Octylisosorbide**

*R*_f_ = 0.38.

ESI–MS: *m*/*z* = 259.26 (*M* + H)^+^, 296.08 (*M* + K)^+^.

^1^H NMR (400 MHz, chloro­form-*d*) δ (ppm) = 4.60 (*t*, *J* = 5.0 Hz, 1H, H4), 4.45 (*d*, *J* = 4.5 Hz, 1H, H3), 4.27 (*dq*, *J* = 7.2 Hz, 5.7 Hz, 1H, H5), 4.06–3.95 (*m*, 2H, H2, H1a), 3.91–3.81 (*m*, 2H, H6, H1b), 3.57 (*dd*, *J* = 9.5 Hz, 5.7 Hz, 1H, H6b), 3.48 (*td*, *J* = 6.7Hz, 2.0 Hz, 2H, H7), 2.64 (*d*, *J* = 7.1 Hz, 1H, OH), 1.56 (*d*, *J* = 11.0 Hz, 2H, H8), 1.35–1.23 (*m*, 10H, H9–H13), 0.88 (*t*, *J* = 6.7 Hz, 3H, H14).

^13^C NMR (101 MHz, chloro­form-*d*) δ (ppm) = 86.1 (C3), 84.3 (C1), 81.9 (C4), 73.8 (C6), 73.7 (C2), 72.4 (C5), 70.1 (C7), 32.0 (C10), 29.9 (C8), 29.5 (C9), 29.4 (C11), 26.2 (C12), 22.8 (C13), 14.2 (C14).

**2-** ***O*** **-Decyl­isosorbide**

*R*_f_ = 0.41.

ESI–MS: *m*/*z* = 287.22 (*M* + H)^+^, 309.21 (*M* + Na)^+^.

^1^H NMR (400 MHz, chloro­form-*d*) δ (ppm) = 4.61 (*t*, *J* = 5.0 Hz, 1H, H4), 4.45 (*d*, *J* = 4.6 Hz, 1H, H3), 4.27 (*m*, 1H, H5), 4.06–3.96 (*m*, 2H, H2, H1a), 3.88 (*dd*, *J* = 9.9 Hz, 3.7 Hz, 1H, H1b), 3.85 (*dd*, *J* = 10.0Hz, 6.3 Hz, 1H, H6a), 3.57 (*dd*, *J* = 9.4 Hz, 5.7 Hz, 1H, H6b), 3.48 (*td*, *J* = 6.6 Hz, 2.0 Hz, 2H, H7), 2.64 (*d*, *J* = 7.1 Hz, 1H, OH), 1.58–1.52 (*m*, 2H, H8), 1.35–1.17 (*m*, 14H, H9–H15), 0.88 (*t*, *J* = 6.7Hz, 3H, H16).

^13^C NMR (101 MHz, chloro­form-*d*) δ (ppm) = 86.1 (C3), 84.2 (C1), 81.8 (C4), 73.8 (C6), 73.6 (C2), 72.4 (C5), 70.1 (C7), 32.0–22.8 (C8–C15), 14.3 (C16).

**2-** ***O*** **-Do­decyl­isosorbide**

*R*_f_ = 0.56.

ESI–MS: *m*/*z* = 315.25 (*M* + H)^+^, 337.24 (*M* + Na)^+^.

m.p. = 327.2–328.7 K.

^1^H NMR (400 MHz, chloro­form–*d*) δ (ppm) = 4.60 (*t*, *J* = 5.0 Hz, 1H, H4), 4.44 (*d*, *J* = 4.5 Hz, 1H, H3), 4.27 (*m*, 1H, H5), 4.04–3.93 (*m*, 2H, H2, H1a), 3.87 (*dd*, *J* = 10.0, 3.6 Hz, 1H, H1b), 3.84 (*dd*, *J* = 9.9 Hz, 6.3 Hz, 1H, H6a), 3.56 (*dd*, *J* = 9.4 Hz, 5.7 Hz, 1H, H6b), 3.47 (*td*, *J* = 6.7 Hz, 2.0 Hz, 2H, H7), 2.67 (*d*, *J* = 7.0 Hz, 1H, OH), 1.61–1.49 (*m*, 2H, H8), 1.25 (*s*, 18H, H9–H17), 0.87 (*t*, *J* = 6.7 Hz, 3H, H18).

^13^C NMR (101 MHz, chloro­form-*d*) δ (ppm) = 86.1 (C3), 84.2 (C1), 81.8 (C4), 73.7 (C6), 73.6 (C2), 72.4 (C5), 70.1 (C7), 32.1–22.8 (C8–C17), 14.3 (C18).

**2-** ***O*** **-Tetra­decyl­isosorbide**

*R*_f_ = 0.69.

ESI–MS: *m*/z = 343.28 (*M* + Na)^+^, 365.27 (*M* + Na)^+^.

^1^H NMR (400 MHz, chloro­form–*d*) δ (ppm) = 4.60 (*t*, *J* = 4.9 Hz, 1H, H4), 4.45 (*d*, *J* = 4.5 Hz, 1H, H3), 4.27 (*dq*, *J* = 7.2 Hz, 5.7 Hz, 1H, H5), 4.04–3.97 (*m*, 2H, H2, H1a), 3.89 (*dd*, *J* = 9.9 Hz, 3.9 Hz, 1H, H1b), 3.85 (*dd*, *J* = 9.5Hz, 5.9 Hz, 1H, H6a), 3.57 (*dd*, *J* = 9.4 Hz, 5.6 Hz, 1H, H6b), 3.48 (*td*, *J* = 6.7 Hz, 3.0 Hz, 2H, H7), 2.65 (*d*, *J* = 7.1 Hz, 1H, OH), 1.58–1.52 (*m*, 2H, H8), 1.35–1.17 (*m*, 14H, H9–H19), 0.88 (*t*, *J* = 6.9 Hz, 3H, H20).

^13^C NMR (101MHz, chloro­form-*d*) δ (ppm) = 86.1 (C3), 84.2 (C1), 81.8 (C4), 73.7 (C6), 73.6 (C2), 72.4 (C5), 70.1 (C7), 32.1–22.8 (C8–C19), 14.3 (C20).

## Refinement   

Crystal data, data collection and structure refinement details are summarized in Table 3 ▸. Methyl groups were refined as idealized rigid groups allowed to rotate but not tip (C—H = 0.98 Å and H—C—H = 109.5°). Other hydrogen atoms were included using a riding model starting from calculated positions (methyl­ene C—H = 0.98 and methine C—H = 1.00 Å). The *U*_iso_(H) values were fixed at 1.5 (for the methyl H and hy­droxy H) or 1.2 times the equivalent U_iso_ value of the parent carbon atoms and oxygen atom, respectively.

## Supplementary Material

Crystal structure: contains datablock(s) iso-c8, iso-c10, iso-c12, iso-c14. DOI: 10.1107/S2056989020006647/zq2253sup1.cif

Supporting information file. DOI: 10.1107/S2056989020006647/zq2253Iso-C8sup6.cdx

Supporting information file. DOI: 10.1107/S2056989020006647/zq2253iso-c8sup10.cml

Structure factors: contains datablock(s) iso-c8. DOI: 10.1107/S2056989020006647/zq2253iso-c8sup13.hkl

Supporting information file. DOI: 10.1107/S2056989020006647/zq2253Iso-C10sup7.cdx

Supporting information file. DOI: 10.1107/S2056989020006647/zq2253iso-c10sup11.cml

Structure factors: contains datablock(s) iso-c10. DOI: 10.1107/S2056989020006647/zq2253iso-c10sup14.hkl

Supporting information file. DOI: 10.1107/S2056989020006647/zq2253Iso-C12sup8.cdx

Supporting information file. DOI: 10.1107/S2056989020006647/zq2253iso-c12sup12.cml

Structure factors: contains datablock(s) iso-c12. DOI: 10.1107/S2056989020006647/zq2253iso-c12sup15.hkl

Supporting information file. DOI: 10.1107/S2056989020006647/zq2253Iso-C14sup9.cdx

Supporting information file. DOI: 10.1107/S2056989020006647/zq2253iso-c14sup13.cml

Structure factors: contains datablock(s) iso-c14. DOI: 10.1107/S2056989020006647/zq2253iso-c14sup16.hkl

CCDC references: 2004430, 2004429, 2004428, 2004427

Additional supporting information:  crystallographic information; 3D view; checkCIF report

## Figures and Tables

**Figure 4:**
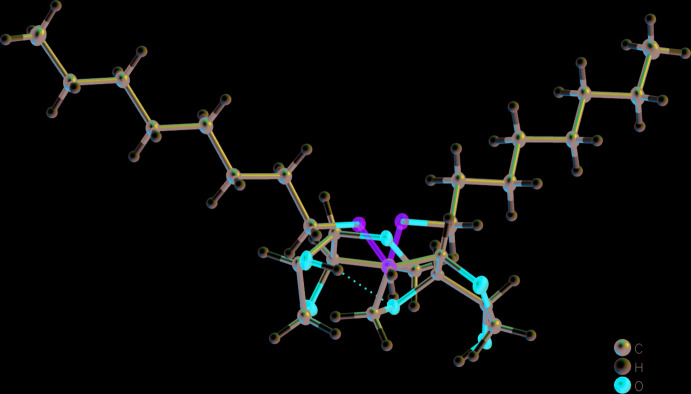
Detail of the packing diagram of **3a** with the inter­molecular torsion angle highlighted in green. The inter­molecular torsion angle corresponds to the opening angle of the herringbone pattern. Ellipsoids represent 50% probability levels.

**Table 1:** Selected geometry parameters and inter­molecular torsion angles (Å, °)

Compound	**3a** Iso-C_8_	**3b** Iso-C_10_	**3c** Iso-C_12_	**3d** Iso-C_14_
C2—C3—O3	111.02 (18)	111.40 (19)	110.8 (3)	111.3 (3)
O1—C4—C5	110.78 (18)	111.05 (19)	110.8 (3)	110.5 (3)
O2—C2	1.422 (3)	1.422 (3)	1.424 (5)	1.430 (4)
Torsion angle O2—C2⋯C2—O2^i^	52.375	53.870	53.646	54.854

Symmetry code: (i) −*x*, *y* + 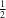, −*z* −  for **3a** and −*x*, *y* − 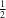, −*z* + 1 for **3b**, **3c** and **3d**.

**Table 2:** Hydrogen-bond geometry (Å,°)

Compound	**3a** Iso-C_8_	**3b** Iso-C_10_	**3c** Iso-C_12_	**3d** Iso-C_14_
O4—H4	0.88 (3)	0.89 (4)	0.90 (7)	0.83 (4)
H4⋯O1^i^	2.00 (3)	1.97 (5)	1.96 (7)	2.03 (4)
O4⋯O1^i^	2.827 (2)	2.823 (3)	2.830 (4)	2.834 (4)
O4—H4⋯O1^i^	155 (3)	160 (4)	162 (5)	161 (4)

Symmetry code: (i) −*x*, *y* + 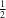, −*z* −  for **3a** and −*x*, *y* − 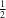, −*z* + 1 for **3b**, **3c** and **3d**.

**Table 3:** Experimental details

	Iso-C_8_	Iso-C_10_	Iso-C_12_	Iso-C_14_
Crystal data				
Chemical formula	C_14_H_26_O_4_	C_16_H_30_O_4_	C_18_H_34_O_4_	C_20_H_38_O_4_
*M*_r_	258.35	286.40	314.45	342.50
Crystal system, space group	Monoclinic, *P*2_1_	Monoclinic, *P*2_1_	Monoclinic, *P*2_1_	Monoclinic, *P*2_1_
Temperature (K)	100	100	100	100
*a*, *b*, *c* (Å)	7.0008 (13), 5.5112 (10), 18.544 (3)	6.9892 (2), 5.4888 (2), 20.8041 (6)	7.0250 (5), 5.4674 (5), 23.377 (2)	7.040 (6), 5.438 (5), 25.56 (2)
β (°)	100.155 (4)	91.302 (3)	97.051 (9)	91.914 (9)
*V* (Å^3^)	704.3 (2)	797.89 (4)	891.08 (14)	978.2 (14)
*Z*	2	2	2	2
Radiation type	Mo *K*α	Cu *K*α	Mo *K*α	Mo *K*α
μ (mm^−1^)	0.09	0.67	0.08	0.08
Crystal size (mm)	0.29 × 0.15 × 0.05	0.44 × 0.16 × 0.08	0.37 × 0.08 × 0.03	0.3 × 0.1 × 0.02

Data collection				
Diffractometer	Bruker APEXII CCD	Rigaku Oxford Diffraction SuperNova, Dual, Atlas	Rigaku Oxford Diffraction SuperNova, Dual, Atlas	Bruker APEXII CCD
Absorption correction	Numerical (*SADABS*; Bruker, 2016 ▸)	Multi-scan (*CrysAlis PRO*; Rigaku OD, 2020 ▸)	Multi-scan (*CrysAlis PRO*; Rigaku OD, 2020 ▸)	Numerical (*SADABS*; Bruker, 2016 ▸)
*T*_min_, *T*_max_	0.604, 0.746	0.590, 1.000	0.534, 1.000	0.543, 0.746
No. of measured, independent and observed [*I* > 2σ(*I*)] reflections	16451, 3491, 3128	17428, 3268, 3028	20988, 4546, 3638	12041, 4278, 2949
*R*_int_	0.048	0.048	0.089	0.069
(sin θ/λ)_max_ (Å^−1^)	0.668	0.632	0.692	0.640

Refinement				
*R*[*F* ^2^ > 2σ(*F* ^2^)], *wR*(*F* ^2^), *S*	0.042, 0.110, 1.10	0.042, 0.118, 1.07	0.082, 0.202, 1.10	0.055, 0.140, 1.04
No. of reflections	3491	3268	4546	4278
No. of parameters	167	185	203	221
No. of restraints	1	1	1	1
H-atom treatment	H atoms treated by a mixture of independent and constrained refinement	H atoms treated by a mixture of independent and constrained refinement	H atoms treated by a mixture of independent and constrained refinement	H atoms treated by a mixture of independent and constrained refinement
Δρ_max_, Δρ_min_ (e Å^−3^)	0.29, −0.22	0.28, −0.21	0.50, −0.38	0.22, −0.24
Absolute structure	Flack *x* determined using 1274 quotients [(*I* ^+^)−(*I* ^−^)]/[(*I* ^+^)+(*I* ^−^)] (Parsons *et al.*, 2013 ▸)	Flack *x* determined using 1249 quotients [(*I* ^+^)−(*I* ^−^)]/[(*I* ^+^)+(*I* ^−^)] (Parsons *et al.*, 2013 ▸)	Flack *x* determined using 1143 quotients [(*I* ^+^)−(*I* ^−^)]/[(*I* ^+^)+(*I* ^−^)] (Parsons *et al.*, 2013 ▸)	Flack *x* determined using 980 quotients [(*I* ^+^)−(*I* ^−^)]/[(*I* ^+^)+(*I* ^−^)] (Parsons *et al.*, 2013 ▸)
Absolute structure parameter	−0.7 (5)	−0.18 (13)	−0.6 (9)	−1.2 (10)

Computer programs: *CrysAlis PRO* (Rigaku OD, 2020 ▸), *BlS* (Bruker, 2016 ▸), *SAINT* (Bruker, 2019 ▸), *SHELXT2014/5* and *SHELXT2018/2* (Sheldrick, 2015*a* ▸), *SHELXL2018/3* (Sheldrick, 2015*b* ▸) and *OLEX2* (Dolomanov *et al.*, 2009 ▸).

## supplementary crystallographic information

### 6-(Octyloxy)hexahydrofuro[3,2-b]furan-3-ol (iso-c8) Crystal data

**Table:**

C_14_H_26_O_4_	*F*(000) = 284
*M**_r_* = 258.35	*D*_x_ = 1.218 Mg m^−^^3^
Monoclinic, *P*2_1_	Mo *K*α radiation, λ = 0.71073 Å
*a* = 7.0008 (13) Å	Cell parameters from 5399 reflections
*b* = 5.5112 (10) Å	θ = 3.0–27.7°
*c* = 18.544 (3) Å	µ = 0.09 mm^−^^1^
β = 100.155 (4)°	*T* = 100 K
*V* = 704.3 (2) Å^3^	Plate, colourless
*Z* = 2	0.29 × 0.14 × 0.05 mm

### 6-(Octyloxy)hexahydrofuro[3,2-b]furan-3-ol (iso-c8) Data collection

**Table:**

Bruker APEXII CCD diffractometer	3128 reflections with *I* > 2σ(*I*)
Detector resolution: 8.3 pixels mm^-1^	*R*_int_ = 0.048
φ and ω scans	θ_max_ = 28.3°, θ_min_ = 2.2°
Absorption correction: numerical (SADABS; Bruker, 2016)	*h* = −9→9
*T*_min_ = 0.604, *T*_max_ = 0.746	*k* = −7→7
16451 measured reflections	*l* = −24→24
3491 independent reflections	

### 6-(Octyloxy)hexahydrofuro[3,2-b]furan-3-ol (iso-c8) Refinement

**Table:**

Refinement on *F*^2^	Hydrogen site location: mixed
Least-squares matrix: full	H atoms treated by a mixture of independent and constrained refinement
*R*[*F*^2^ > 2σ(*F*^2^)] = 0.042	*w* = 1/[σ^2^(*F*_o_^2^) + (0.0584*P*)^2^ + 0.0928*P*] where *P* = (*F*_o_^2^ + 2*F*_c_^2^)/3
*wR*(*F*^2^) = 0.110	(Δ/σ)_max_ < 0.001
*S* = 1.10	Δρ_max_ = 0.29 e Å^−^^3^
3491 reflections	Δρ_min_ = −0.22 e Å^−^^3^
167 parameters	Absolute structure: Flack *x* determined using 1274 quotients [(*I*^+^)-(*I*^-^)]/[(*I*^+^)+(*I*^-^)] (Parsons *et al.*, 2013)
1 restraint	Absolute structure parameter: −0.7 (5)
Primary atom site location: dual	

### 6-(Octyloxy)hexahydrofuro[3,2-b]furan-3-ol (iso-c8) Special details

**Table:**

Geometry. All esds (except the esd in the dihedral angle between two l.s. planes) are estimated using the full covariance matrix. The cell esds are taken into account individually in the estimation of esds in distances, angles and torsion angles; correlations between esds in cell parameters are only used when they are defined by crystal symmetry. An approximate (isotropic) treatment of cell esds is used for estimating esds involving l.s. planes.

### 6-(Octyloxy)hexahydrofuro[3,2-b]furan-3-ol (iso-c8) Fractional atomic coordinates and isotropic or equivalent isotropic displacement parameters (Å^2^)

**Table:**

	*x*	*y*	*z*	*U*_iso_*/*U*_eq_	
O4	−0.3146 (2)	1.1240 (3)	0.46449 (9)	0.0206 (4)	
H4	−0.204 (4)	1.202 (6)	0.4671 (15)	0.031*	
O3	−0.2626 (2)	1.1015 (3)	0.66173 (9)	0.0223 (4)	
C6	−0.3112 (3)	1.2324 (5)	0.59462 (13)	0.0202 (5)	
H6A	−0.211534	1.356261	0.590200	0.024*	
H6B	−0.438754	1.313362	0.591209	0.024*	
C5	−0.3178 (3)	1.0391 (4)	0.53597 (12)	0.0175 (5)	
H5	−0.439886	0.943600	0.534571	0.021*	
C4	−0.1475 (3)	0.8769 (4)	0.56869 (12)	0.0162 (4)	
H4A	−0.167151	0.705588	0.551189	0.019*	
O1	0.0336 (2)	0.9757 (3)	0.55443 (8)	0.0173 (3)	
C1	0.1401 (3)	1.0801 (4)	0.62113 (12)	0.0184 (5)	
H1A	0.112653	1.255842	0.623537	0.022*	
H1B	0.281444	1.057319	0.623838	0.022*	
C2	0.0702 (3)	0.9457 (4)	0.68292 (12)	0.0178 (5)	
H2	0.083366	1.047420	0.728187	0.021*	
C3	−0.1426 (3)	0.8969 (4)	0.65068 (12)	0.0172 (4)	
H3	−0.189743	0.744399	0.670946	0.021*	
O2	0.1583 (2)	0.7144 (3)	0.69783 (9)	0.0196 (4)	
C7	0.3524 (3)	0.7307 (5)	0.73806 (13)	0.0224 (5)	
H7A	0.355169	0.837569	0.781134	0.027*	
H7B	0.438704	0.801247	0.706650	0.027*	
C8	0.4223 (3)	0.4806 (5)	0.76282 (13)	0.0207 (5)	
H8A	0.421057	0.375583	0.719426	0.025*	
H8B	0.332855	0.409170	0.792837	0.025*	
C9	0.6279 (3)	0.4879 (5)	0.80782 (13)	0.0217 (5)	
H9A	0.716491	0.559354	0.777433	0.026*	
H9B	0.628485	0.595407	0.850628	0.026*	
C10	0.7051 (3)	0.2392 (5)	0.83484 (12)	0.0197 (5)	
H10A	0.625967	0.175594	0.869914	0.024*	
H10B	0.691287	0.126099	0.792767	0.024*	
C11	0.9172 (3)	0.2475 (5)	0.87199 (13)	0.0207 (5)	
H11A	0.994196	0.323853	0.838152	0.025*	
H11B	0.928797	0.351471	0.916094	0.025*	
C12	1.0032 (3)	−0.0002 (4)	0.89449 (13)	0.0205 (5)	
H12A	0.997027	−0.102242	0.850206	0.025*	
H12B	0.923641	−0.079522	0.926877	0.025*	
C13	1.2132 (3)	0.0131 (5)	0.93423 (13)	0.0234 (5)	
H13A	1.290702	0.102721	0.903187	0.028*	
H13B	1.217835	0.106071	0.980190	0.028*	
C14	1.3052 (4)	−0.2343 (5)	0.95257 (15)	0.0286 (6)	
H14A	1.306695	−0.324955	0.907209	0.043*	
H14B	1.229830	−0.324164	0.983527	0.043*	
H14C	1.438497	−0.213184	0.978793	0.043*	

### 6-(Octyloxy)hexahydrofuro[3,2-b]furan-3-ol (iso-c8) Atomic displacement parameters (Å^2^)

**Table:**

	*U*^11^	*U*^22^	*U*^33^	*U*^12^	*U*^13^	*U*^23^
O4	0.0132 (7)	0.0254 (9)	0.0228 (8)	−0.0035 (7)	0.0025 (6)	0.0041 (7)
O3	0.0213 (8)	0.0237 (9)	0.0231 (9)	0.0053 (7)	0.0074 (6)	−0.0009 (7)
C6	0.0159 (10)	0.0189 (11)	0.0264 (12)	0.0016 (9)	0.0056 (8)	0.0013 (10)
C5	0.0129 (9)	0.0176 (11)	0.0223 (11)	−0.0009 (8)	0.0040 (8)	0.0014 (9)
C4	0.0142 (10)	0.0113 (9)	0.0237 (11)	−0.0013 (8)	0.0053 (8)	0.0014 (8)
O1	0.0127 (7)	0.0192 (8)	0.0208 (8)	−0.0004 (6)	0.0052 (6)	0.0002 (6)
C1	0.0154 (10)	0.0162 (10)	0.0234 (11)	−0.0010 (8)	0.0030 (8)	−0.0009 (9)
C2	0.0169 (10)	0.0164 (11)	0.0198 (11)	−0.0006 (8)	0.0026 (8)	−0.0004 (9)
C3	0.0139 (10)	0.0139 (10)	0.0243 (12)	−0.0002 (8)	0.0045 (8)	0.0026 (9)
O2	0.0151 (7)	0.0172 (8)	0.0251 (9)	0.0001 (7)	−0.0002 (6)	0.0024 (7)
C7	0.0152 (10)	0.0240 (12)	0.0267 (12)	−0.0029 (10)	−0.0004 (8)	0.0034 (11)
C8	0.0157 (10)	0.0209 (11)	0.0244 (12)	−0.0006 (9)	0.0008 (8)	0.0020 (10)
C9	0.0160 (10)	0.0220 (11)	0.0260 (12)	−0.0025 (9)	0.0008 (8)	0.0028 (10)
C10	0.0155 (10)	0.0222 (11)	0.0211 (11)	−0.0011 (9)	0.0022 (8)	0.0006 (10)
C11	0.0154 (10)	0.0217 (11)	0.0252 (12)	−0.0005 (9)	0.0039 (8)	0.0011 (10)
C12	0.0159 (10)	0.0215 (11)	0.0242 (12)	−0.0005 (9)	0.0039 (8)	0.0010 (10)
C13	0.0154 (10)	0.0255 (13)	0.0287 (13)	0.0008 (9)	0.0023 (8)	0.0019 (10)
C14	0.0220 (12)	0.0317 (15)	0.0311 (14)	0.0053 (10)	0.0024 (10)	0.0023 (11)

### 6-(Octyloxy)hexahydrofuro[3,2-b]furan-3-ol (iso-c8) Geometric parameters (Å, º)

**Table:**

O4—H4	0.88 (3)	C7—C8	1.507 (3)
O4—C5	1.410 (3)	C8—H8A	0.9900
O3—C6	1.427 (3)	C8—H8B	0.9900
O3—C3	1.442 (3)	C8—C9	1.532 (3)
C6—H6A	0.9900	C9—H9A	0.9900
C6—H6B	0.9900	C9—H9B	0.9900
C6—C5	1.517 (3)	C9—C10	1.525 (3)
C5—H5	1.0000	C10—H10A	0.9900
C5—C4	1.527 (3)	C10—H10B	0.9900
C4—H4A	1.0000	C10—C11	1.524 (3)
C4—O1	1.447 (2)	C11—H11A	0.9900
C4—C3	1.519 (3)	C11—H11B	0.9900
O1—C1	1.446 (3)	C11—C12	1.520 (3)
C1—H1A	0.9900	C12—H12A	0.9900
C1—H1B	0.9900	C12—H12B	0.9900
C1—C2	1.516 (3)	C12—C13	1.526 (3)
C2—H2	1.0000	C13—H13A	0.9900
C2—C3	1.528 (3)	C13—H13B	0.9900
C2—O2	1.422 (3)	C13—C14	1.521 (4)
C3—H3	1.0000	C14—H14A	0.9800
O2—C7	1.433 (3)	C14—H14B	0.9800
C7—H7A	0.9900	C14—H14C	0.9800
C7—H7B	0.9900		
			
C5—O4—H4	105.8 (19)	C8—C7—H7A	109.8
C6—O3—C3	109.08 (16)	C8—C7—H7B	109.8
O3—C6—H6A	110.9	C7—C8—H8A	109.3
O3—C6—H6B	110.9	C7—C8—H8B	109.3
O3—C6—C5	104.04 (19)	C7—C8—C9	111.45 (19)
H6A—C6—H6B	109.0	H8A—C8—H8B	108.0
C5—C6—H6A	110.9	C9—C8—H8A	109.3
C5—C6—H6B	110.9	C9—C8—H8B	109.3
O4—C5—C6	115.94 (19)	C8—C9—H9A	108.9
O4—C5—H5	107.8	C8—C9—H9B	108.9
O4—C5—C4	115.27 (17)	H9A—C9—H9B	107.7
C6—C5—H5	107.8	C10—C9—C8	113.56 (19)
C6—C5—C4	101.72 (17)	C10—C9—H9A	108.9
C4—C5—H5	107.8	C10—C9—H9B	108.9
C5—C4—H4A	111.8	C9—C10—H10A	109.1
O1—C4—C5	110.80 (18)	C9—C10—H10B	109.1
O1—C4—H4A	111.8	H10A—C10—H10B	107.8
O1—C4—C3	106.84 (17)	C11—C10—C9	112.47 (19)
C3—C4—C5	103.52 (17)	C11—C10—H10A	109.1
C3—C4—H4A	111.8	C11—C10—H10B	109.1
C1—O1—C4	109.25 (16)	C10—C11—H11A	108.8
O1—C1—H1A	110.7	C10—C11—H11B	108.8
O1—C1—H1B	110.7	H11A—C11—H11B	107.7
O1—C1—C2	105.42 (18)	C12—C11—C10	113.8 (2)
H1A—C1—H1B	108.8	C12—C11—H11A	108.8
C2—C1—H1A	110.7	C12—C11—H11B	108.8
C2—C1—H1B	110.7	C11—C12—H12A	109.0
C1—C2—H2	111.5	C11—C12—H12B	109.0
C1—C2—C3	102.28 (18)	C11—C12—C13	113.0 (2)
C3—C2—H2	111.5	H12A—C12—H12B	107.8
O2—C2—C1	113.45 (18)	C13—C12—H12A	109.0
O2—C2—H2	111.5	C13—C12—H12B	109.0
O2—C2—C3	106.12 (17)	C12—C13—H13A	108.9
O3—C3—C4	106.73 (17)	C12—C13—H13B	108.9
O3—C3—C2	111.02 (18)	H13A—C13—H13B	107.7
O3—C3—H3	111.4	C14—C13—C12	113.5 (2)
C4—C3—C2	104.65 (17)	C14—C13—H13A	108.9
C4—C3—H3	111.4	C14—C13—H13B	108.9
C2—C3—H3	111.4	C13—C14—H14A	109.5
C2—O2—C7	112.60 (18)	C13—C14—H14B	109.5
O2—C7—H7A	109.8	C13—C14—H14C	109.5
O2—C7—H7B	109.8	H14A—C14—H14B	109.5
O2—C7—C8	109.21 (19)	H14A—C14—H14C	109.5
H7A—C7—H7B	108.3	H14B—C14—H14C	109.5
			
O4—C5—C4—O1	44.0 (3)	C1—C2—C3—O3	−85.1 (2)
O4—C5—C4—C3	158.17 (18)	C1—C2—C3—C4	29.6 (2)
O3—C6—C5—O4	−164.56 (16)	C1—C2—O2—C7	76.8 (2)
O3—C6—C5—C4	−38.7 (2)	C2—O2—C7—C8	170.27 (18)
C6—O3—C3—C4	−10.3 (2)	C3—O3—C6—C5	31.1 (2)
C6—O3—C3—C2	103.2 (2)	C3—C4—O1—C1	−6.4 (2)
C6—C5—C4—O1	−82.3 (2)	C3—C2—O2—C7	−171.63 (17)
C6—C5—C4—C3	31.9 (2)	O2—C2—C3—O3	155.71 (17)
C5—C4—O1—C1	105.7 (2)	O2—C2—C3—C4	−89.5 (2)
C5—C4—C3—O3	−14.4 (2)	O2—C7—C8—C9	−178.64 (18)
C5—C4—C3—C2	−132.20 (18)	C7—C8—C9—C10	179.5 (2)
C4—O1—C1—C2	25.7 (2)	C8—C9—C10—C11	173.59 (19)
O1—C4—C3—O3	102.57 (19)	C9—C10—C11—C12	−175.6 (2)
O1—C4—C3—C2	−15.2 (2)	C10—C11—C12—C13	−177.75 (19)
O1—C1—C2—C3	−34.0 (2)	C11—C12—C13—C14	−176.2 (2)
O1—C1—C2—O2	79.9 (2)		

### 6-(Decyloxy)hexahydrofuro[3,2-b]furan-3-ol (iso-c10) Crystal data

**Table:**

C_16_H_30_O_4_	*F*(000) = 316
*M**_r_* = 286.40	*D*_x_ = 1.192 Mg m^−^^3^
Monoclinic, *P*2_1_	Cu *K*α radiation, λ = 1.54184 Å
*a* = 6.9892 (2) Å	Cell parameters from 7114 reflections
*b* = 5.4888 (2) Å	θ = 4.2–76.2°
*c* = 20.8041 (6) Å	µ = 0.67 mm^−^^1^
β = 91.302 (3)°	*T* = 100 K
*V* = 797.89 (4) Å^3^	Plate, colourless
*Z* = 2	0.44 × 0.16 × 0.08 mm

### 6-(Decyloxy)hexahydrofuro[3,2-b]furan-3-ol (iso-c10) Data collection

**Table:**

Rigaku Oxford Diffraction SuperNova, Dual, Atlas diffractometer	3028 reflections with *I* > 2σ(*I*)
Detector resolution: 10.4127 pixels mm^-1^	*R*_int_ = 0.048
ω scans	θ_max_ = 76.9°, θ_min_ = 4.3°
Absorption correction: multi-scan (CrysAlisPro; Rigaku OD, 2020)	*h* = −8→8
*T*_min_ = 0.590, *T*_max_ = 1.000	*k* = −6→6
17428 measured reflections	*l* = −26→26
3268 independent reflections	

### 6-(Decyloxy)hexahydrofuro[3,2-b]furan-3-ol (iso-c10) Refinement

**Table:**

Refinement on *F*^2^	Hydrogen site location: mixed
Least-squares matrix: full	H atoms treated by a mixture of independent and constrained refinement
*R*[*F*^2^ > 2σ(*F*^2^)] = 0.042	*w* = 1/[σ^2^(*F*_o_^2^) + (0.0741*P*)^2^ + 0.1068*P*] where *P* = (*F*_o_^2^ + 2*F*_c_^2^)/3
*wR*(*F*^2^) = 0.118	(Δ/σ)_max_ < 0.001
*S* = 1.07	Δρ_max_ = 0.28 e Å^−^^3^
3268 reflections	Δρ_min_ = −0.21 e Å^−^^3^
185 parameters	Absolute structure: Flack *x* determined using 1249 quotients [(*I*^+^)-(*I*^-^)]/[(*I*^+^)+(*I*^-^)] (Parsons *et al.*, 2013)
1 restraint	Absolute structure parameter: −0.18 (13)
Primary atom site location: dual	

### 6-(Decyloxy)hexahydrofuro[3,2-b]furan-3-ol (iso-c10) Special details

**Table:**

Geometry. All esds (except the esd in the dihedral angle between two l.s. planes) are estimated using the full covariance matrix. The cell esds are taken into account individually in the estimation of esds in distances, angles and torsion angles; correlations between esds in cell parameters are only used when they are defined by crystal symmetry. An approximate (isotropic) treatment of cell esds is used for estimating esds involving l.s. planes.

### 6-(Decyloxy)hexahydrofuro[3,2-b]furan-3-ol (iso-c10) Fractional atomic coordinates and isotropic or equivalent isotropic displacement parameters (Å^2^)

**Table:**

	*x*	*y*	*z*	*U*_iso_*/*U*_eq_	
O4	−0.2948 (2)	−0.2388 (4)	0.53094 (8)	0.0285 (4)	
C5	−0.3362 (3)	−0.1528 (5)	0.46833 (11)	0.0261 (5)	
H5	−0.457030	−0.055647	0.469645	0.031*	
C6	−0.3615 (4)	−0.3478 (5)	0.41677 (12)	0.0285 (5)	
H6A	−0.259150	−0.472001	0.420463	0.034*	
H6B	−0.487258	−0.429298	0.420144	0.034*	
O3	−0.3497 (3)	−0.2169 (4)	0.35754 (8)	0.0295 (4)	
C3	−0.2217 (3)	−0.0132 (4)	0.36691 (11)	0.0253 (5)	
H3	−0.279228	0.140392	0.349299	0.030*	
C4	−0.1813 (3)	0.0076 (4)	0.43919 (11)	0.0236 (5)	
H4A	−0.189557	0.179951	0.454433	0.028*	
O1	0.0067 (2)	−0.0944 (3)	0.45089 (8)	0.0260 (4)	
C1	0.0779 (3)	−0.1993 (4)	0.39222 (11)	0.0271 (5)	
H1A	0.049214	−0.375810	0.390087	0.032*	
H1B	0.217929	−0.176099	0.389610	0.032*	
C2	−0.0268 (3)	−0.0629 (4)	0.33802 (11)	0.0253 (5)	
H2	−0.039074	−0.165289	0.298387	0.030*	
O2	0.0557 (3)	0.1676 (3)	0.32444 (8)	0.0274 (4)	
C7	0.2266 (4)	0.1490 (5)	0.28830 (12)	0.0300 (5)	
H7A	0.203111	0.045168	0.250013	0.036*	
H7B	0.329320	0.072848	0.314992	0.036*	
C8	0.2878 (4)	0.4002 (5)	0.26759 (12)	0.0278 (5)	
H8A	0.310745	0.502828	0.306136	0.033*	
H8B	0.183564	0.475990	0.241529	0.033*	
C9	0.4700 (4)	0.3917 (5)	0.22816 (12)	0.0282 (5)	
H9A	0.574428	0.319412	0.254840	0.034*	
H9B	0.447666	0.284008	0.190571	0.034*	
C10	0.5339 (4)	0.6413 (5)	0.20451 (11)	0.0266 (5)	
H10A	0.546751	0.753028	0.241709	0.032*	
H10B	0.434015	0.708219	0.174967	0.032*	
C11	0.7238 (4)	0.6321 (5)	0.16973 (12)	0.0283 (5)	
H11A	0.821209	0.554476	0.198301	0.034*	
H11B	0.708077	0.528647	0.130987	0.034*	
C12	0.7967 (4)	0.8820 (5)	0.14951 (12)	0.0282 (5)	
H12A	0.813283	0.985547	0.188208	0.034*	
H12B	0.699513	0.960098	0.120955	0.034*	
C13	0.9863 (4)	0.8696 (5)	0.11464 (11)	0.0277 (5)	
H13A	1.082709	0.789157	0.143048	0.033*	
H13B	0.969014	0.767306	0.075754	0.033*	
C14	1.0631 (4)	1.1179 (5)	0.09472 (12)	0.0282 (5)	
H14A	0.963902	1.202929	0.068427	0.034*	
H14B	1.088498	1.216908	0.133771	0.034*	
C15	1.2459 (4)	1.1010 (5)	0.05646 (12)	0.0305 (6)	
H15A	1.218905	1.007541	0.016575	0.037*	
H15B	1.343110	1.009609	0.082066	0.037*	
C16	1.3286 (4)	1.3485 (5)	0.03860 (13)	0.0346 (6)	
H16A	1.233105	1.440762	0.013414	0.052*	
H16B	1.443206	1.324933	0.013041	0.052*	
H16C	1.362570	1.438813	0.077856	0.052*	
H4	−0.190 (6)	−0.330 (8)	0.5295 (18)	0.052*	

### 6-(Decyloxy)hexahydrofuro[3,2-b]furan-3-ol (iso-c10) Atomic displacement parameters (Å^2^)

**Table:**

	*U*^11^	*U*^22^	*U*^33^	*U*^12^	*U*^13^	*U*^23^
O4	0.0223 (8)	0.0372 (9)	0.0258 (8)	0.0032 (8)	−0.0004 (6)	0.0043 (7)
C5	0.0219 (11)	0.0299 (12)	0.0262 (11)	0.0040 (9)	−0.0035 (9)	0.0016 (9)
C6	0.0253 (12)	0.0288 (11)	0.0312 (12)	−0.0026 (10)	−0.0021 (9)	0.0011 (10)
O3	0.0263 (9)	0.0364 (9)	0.0256 (8)	−0.0065 (7)	−0.0063 (6)	0.0005 (7)
C3	0.0208 (11)	0.0293 (11)	0.0256 (11)	−0.0001 (9)	−0.0046 (9)	0.0006 (9)
C4	0.0216 (11)	0.0232 (10)	0.0257 (10)	0.0045 (9)	−0.0040 (8)	0.0003 (9)
O1	0.0209 (8)	0.0315 (8)	0.0255 (8)	0.0018 (7)	−0.0049 (6)	0.0012 (6)
C1	0.0237 (11)	0.0293 (11)	0.0281 (11)	0.0021 (9)	−0.0021 (9)	0.0007 (9)
C2	0.0233 (12)	0.0286 (12)	0.0238 (10)	0.0005 (9)	−0.0020 (9)	−0.0007 (9)
O2	0.0249 (9)	0.0290 (8)	0.0284 (8)	−0.0002 (7)	0.0019 (7)	0.0017 (7)
C7	0.0255 (12)	0.0330 (12)	0.0316 (12)	0.0028 (10)	0.0030 (10)	0.0030 (10)
C8	0.0250 (12)	0.0313 (12)	0.0271 (11)	0.0019 (10)	0.0009 (9)	0.0009 (9)
C9	0.0246 (12)	0.0329 (12)	0.0270 (11)	0.0022 (10)	0.0005 (9)	0.0033 (10)
C10	0.0228 (12)	0.0322 (11)	0.0247 (10)	0.0011 (10)	−0.0022 (8)	0.0008 (9)
C11	0.0229 (12)	0.0324 (12)	0.0296 (11)	0.0007 (10)	−0.0015 (9)	0.0020 (10)
C12	0.0231 (12)	0.0319 (11)	0.0294 (11)	0.0010 (9)	0.0001 (9)	0.0012 (10)
C13	0.0224 (12)	0.0315 (12)	0.0290 (11)	0.0003 (9)	−0.0012 (9)	0.0015 (10)
C14	0.0225 (12)	0.0319 (12)	0.0302 (11)	−0.0008 (10)	−0.0016 (9)	−0.0008 (10)
C15	0.0250 (13)	0.0344 (13)	0.0320 (12)	−0.0007 (10)	−0.0003 (10)	0.0019 (10)
C16	0.0304 (13)	0.0398 (15)	0.0337 (12)	−0.0064 (11)	0.0006 (10)	0.0019 (11)

### 6-(Decyloxy)hexahydrofuro[3,2-b]furan-3-ol (iso-c10) Geometric parameters (Å, º)

**Table:**

O4—C5	1.409 (3)	C8—C9	1.531 (3)
O4—H4	0.89 (4)	C9—H9A	0.9900
C5—H5	1.0000	C9—H9B	0.9900
C5—C6	1.523 (3)	C9—C10	1.526 (3)
C5—C4	1.531 (3)	C10—H10A	0.9900
C6—H6A	0.9900	C10—H10B	0.9900
C6—H6B	0.9900	C10—C11	1.527 (3)
C6—O3	1.430 (3)	C11—H11A	0.9900
O3—C3	1.442 (3)	C11—H11B	0.9900
C3—H3	1.0000	C11—C12	1.526 (3)
C3—C4	1.528 (3)	C12—H12A	0.9900
C3—C2	1.526 (3)	C12—H12B	0.9900
C4—H4A	1.0000	C12—C13	1.527 (3)
C4—O1	1.444 (3)	C13—H13A	0.9900
O1—C1	1.448 (3)	C13—H13B	0.9900
C1—H1A	0.9900	C13—C14	1.525 (3)
C1—H1B	0.9900	C14—H14A	0.9900
C1—C2	1.526 (3)	C14—H14B	0.9900
C2—H2	1.0000	C14—C15	1.524 (3)
C2—O2	1.422 (3)	C15—H15A	0.9900
O2—C7	1.429 (3)	C15—H15B	0.9900
C7—H7A	0.9900	C15—C16	1.526 (4)
C7—H7B	0.9900	C16—H16A	0.9800
C7—C8	1.510 (4)	C16—H16B	0.9800
C8—H8A	0.9900	C16—H16C	0.9800
C8—H8B	0.9900		
			
C5—O4—H4	108 (2)	C9—C8—H8A	109.3
O4—C5—H5	108.0	C9—C8—H8B	109.3
O4—C5—C6	115.7 (2)	C8—C9—H9A	108.9
O4—C5—C4	115.25 (19)	C8—C9—H9B	108.9
C6—C5—H5	108.0	H9A—C9—H9B	107.7
C6—C5—C4	101.37 (18)	C10—C9—C8	113.5 (2)
C4—C5—H5	108.0	C10—C9—H9A	108.9
C5—C6—H6A	110.9	C10—C9—H9B	108.9
C5—C6—H6B	110.9	C9—C10—H10A	109.0
H6A—C6—H6B	108.9	C9—C10—H10B	109.0
O3—C6—C5	104.2 (2)	C9—C10—C11	112.8 (2)
O3—C6—H6A	110.9	H10A—C10—H10B	107.8
O3—C6—H6B	110.9	C11—C10—H10A	109.0
C6—O3—C3	108.71 (18)	C11—C10—H10B	109.0
O3—C3—H3	111.2	C10—C11—H11A	108.8
O3—C3—C4	106.92 (19)	C10—C11—H11B	108.8
O3—C3—C2	111.40 (19)	H11A—C11—H11B	107.7
C4—C3—H3	111.2	C12—C11—C10	113.6 (2)
C2—C3—H3	111.2	C12—C11—H11A	108.8
C2—C3—C4	104.72 (18)	C12—C11—H11B	108.8
C5—C4—H4A	111.8	C11—C12—H12A	109.0
C3—C4—C5	103.31 (19)	C11—C12—H12B	109.0
C3—C4—H4A	111.8	C11—C12—C13	113.0 (2)
O1—C4—C5	111.05 (19)	H12A—C12—H12B	107.8
O1—C4—C3	106.51 (18)	C13—C12—H12A	109.0
O1—C4—H4A	111.8	C13—C12—H12B	109.0
C4—O1—C1	109.92 (17)	C12—C13—H13A	108.8
O1—C1—H1A	110.7	C12—C13—H13B	108.8
O1—C1—H1B	110.7	H13A—C13—H13B	107.7
O1—C1—C2	105.05 (18)	C14—C13—C12	113.8 (2)
H1A—C1—H1B	108.8	C14—C13—H13A	108.8
C2—C1—H1A	110.7	C14—C13—H13B	108.8
C2—C1—H1B	110.7	C13—C14—H14A	109.0
C3—C2—H2	111.4	C13—C14—H14B	109.0
C1—C2—C3	102.34 (18)	H14A—C14—H14B	107.8
C1—C2—H2	111.4	C15—C14—C13	113.1 (2)
O2—C2—C3	106.81 (18)	C15—C14—H14A	109.0
O2—C2—C1	113.20 (19)	C15—C14—H14B	109.0
O2—C2—H2	111.4	C14—C15—H15A	108.9
C2—O2—C7	112.85 (19)	C14—C15—H15B	108.9
O2—C7—H7A	109.8	C14—C15—C16	113.6 (2)
O2—C7—H7B	109.8	H15A—C15—H15B	107.7
O2—C7—C8	109.3 (2)	C16—C15—H15A	108.9
H7A—C7—H7B	108.3	C16—C15—H15B	108.9
C8—C7—H7A	109.8	C15—C16—H16A	109.5
C8—C7—H7B	109.8	C15—C16—H16B	109.5
C7—C8—H8A	109.3	C15—C16—H16C	109.5
C7—C8—H8B	109.3	H16A—C16—H16B	109.5
C7—C8—C9	111.7 (2)	H16A—C16—H16C	109.5
H8A—C8—H8B	107.9	H16B—C16—H16C	109.5
			
O4—C5—C6—O3	−164.89 (19)	C4—C3—C2—O2	−89.3 (2)
O4—C5—C4—C3	157.97 (19)	C4—O1—C1—C2	25.1 (2)
O4—C5—C4—O1	44.1 (3)	O1—C1—C2—C3	−33.7 (2)
C5—C6—O3—C3	31.7 (2)	O1—C1—C2—O2	80.9 (2)
C5—C4—O1—C1	106.0 (2)	C1—C2—O2—C7	76.9 (2)
C6—C5—C4—C3	32.2 (2)	C2—C3—C4—C5	−132.86 (19)
C6—C5—C4—O1	−81.6 (2)	C2—C3—C4—O1	−15.8 (2)
C6—O3—C3—C4	−10.6 (2)	C2—O2—C7—C8	171.6 (2)
C6—O3—C3—C2	103.3 (2)	O2—C7—C8—C9	−179.61 (19)
O3—C3—C4—C5	−14.6 (2)	C7—C8—C9—C10	178.3 (2)
O3—C3—C4—O1	102.5 (2)	C8—C9—C10—C11	175.6 (2)
O3—C3—C2—C1	−85.4 (2)	C9—C10—C11—C12	−176.2 (2)
O3—C3—C2—O2	155.46 (18)	C10—C11—C12—C13	−179.8 (2)
C3—C4—O1—C1	−5.8 (2)	C11—C12—C13—C14	−179.3 (2)
C3—C2—O2—C7	−171.21 (18)	C12—C13—C14—C15	−176.5 (2)
C4—C5—C6—O3	−39.5 (2)	C13—C14—C15—C16	−177.7 (2)
C4—C3—C2—C1	29.9 (2)		

### 6-(Dodecyloxy)hexahydrofuro[3,2-b]furan-3-ol (iso-c12) Crystal data

**Table:**

C_18_H_34_O_4_	*F*(000) = 348
*M**_r_* = 314.45	*D*_x_ = 1.172 Mg m^−^^3^
Monoclinic, *P*2_1_	Mo *K*α radiation, λ = 0.71073 Å
*a* = 7.0250 (5) Å	Cell parameters from 3996 reflections
*b* = 5.4674 (5) Å	θ = 3.5–28.5°
*c* = 23.377 (2) Å	µ = 0.08 mm^−^^1^
β = 97.051 (9)°	*T* = 100 K
*V* = 891.08 (14) Å^3^	Plate, colourless
*Z* = 2	0.37 × 0.08 × 0.03 mm

### 6-(Dodecyloxy)hexahydrofuro[3,2-b]furan-3-ol (iso-c12) Data collection

**Table:**

Rigaku Oxford Diffraction SuperNova, Dual, Atlas diffractometer	3638 reflections with *I* > 2σ(*I*)
Detector resolution: 5.2063 pixels mm^-1^	*R*_int_ = 0.089
ω scans	θ_max_ = 29.5°, θ_min_ = 2.6°
Absorption correction: multi-scan (CrysAlisPro; Rigaku OD, 2020)	*h* = −9→9
*T*_min_ = 0.534, *T*_max_ = 1.000	*k* = −7→7
20988 measured reflections	*l* = −31→31
4546 independent reflections	

### 6-(Dodecyloxy)hexahydrofuro[3,2-b]furan-3-ol (iso-c12) Refinement

**Table:**

Refinement on *F*^2^	Hydrogen site location: mixed
Least-squares matrix: full	H atoms treated by a mixture of independent and constrained refinement
*R*[*F*^2^ > 2σ(*F*^2^)] = 0.082	*w* = 1/[σ^2^(*F*_o_^2^) + (0.0818*P*)^2^ + 0.8491*P*] where *P* = (*F*_o_^2^ + 2*F*_c_^2^)/3
*wR*(*F*^2^) = 0.202	(Δ/σ)_max_ < 0.001
*S* = 1.10	Δρ_max_ = 0.50 e Å^−^^3^
4546 reflections	Δρ_min_ = −0.38 e Å^−^^3^
203 parameters	Absolute structure: Flack *x* determined using 1143 quotients [(*I*^+^)-(*I*^-^)]/[(*I*^+^)+(*I*^-^)] (Parsons *et al.*, 2013)
1 restraint	Absolute structure parameter: −0.6 (9)
Primary atom site location: dual	

### 6-(Dodecyloxy)hexahydrofuro[3,2-b]furan-3-ol (iso-c12) Special details

**Table:**

Geometry. All esds (except the esd in the dihedral angle between two l.s. planes) are estimated using the full covariance matrix. The cell esds are taken into account individually in the estimation of esds in distances, angles and torsion angles; correlations between esds in cell parameters are only used when they are defined by crystal symmetry. An approximate (isotropic) treatment of cell esds is used for estimating esds involving l.s. planes.

### 6-(Dodecyloxy)hexahydrofuro[3,2-b]furan-3-ol (iso-c12) Fractional atomic coordinates and isotropic or equivalent isotropic displacement parameters (Å^2^)

**Table:**

	*x*	*y*	*z*	*U*_iso_*/*U*_eq_	
O4	−0.3077 (4)	−0.3567 (6)	0.47225 (13)	0.0195 (7)	
C5	−0.3215 (5)	−0.2711 (8)	0.52867 (18)	0.0158 (9)	
H5	−0.439260	−0.174098	0.527443	0.019*	
C6	−0.3266 (6)	−0.4653 (9)	0.57474 (19)	0.0174 (9)	
H6A	−0.451597	−0.542957	0.571783	0.021*	
H6B	−0.229956	−0.589455	0.571468	0.021*	
O3	−0.2875 (4)	−0.3345 (7)	0.62790 (13)	0.0208 (7)	
C3	−0.1630 (5)	−0.1300 (8)	0.61918 (19)	0.0159 (9)	
H3	−0.210764	0.021798	0.634561	0.019*	
C4	−0.1536 (5)	−0.1104 (8)	0.55496 (18)	0.0140 (8)	
H4A	−0.167596	0.059104	0.541499	0.017*	
O1	0.0281 (4)	−0.2142 (6)	0.54414 (12)	0.0153 (6)	
C1	0.1227 (5)	−0.3212 (9)	0.59724 (17)	0.0170 (9)	
H1A	0.260627	−0.301348	0.599673	0.020*	
H1B	0.093479	−0.494261	0.599072	0.020*	
C2	0.0441 (5)	−0.1834 (7)	0.64566 (19)	0.0136 (8)	
H2	0.047254	−0.284334	0.680410	0.016*	
O2	0.1320 (4)	0.0484 (6)	0.65820 (13)	0.0166 (7)	
C7	0.3182 (6)	0.0286 (9)	0.6908 (2)	0.0188 (9)	
H7A	0.406758	−0.047907	0.667560	0.023*	
H7B	0.310862	−0.072266	0.724628	0.023*	
C8	0.3891 (6)	0.2810 (8)	0.7090 (2)	0.0166 (9)	
H8A	0.298790	0.357484	0.731649	0.020*	
H8B	0.396366	0.380691	0.674985	0.020*	
C9	0.5881 (6)	0.2697 (9)	0.74473 (19)	0.0175 (9)	
H9A	0.580593	0.166828	0.778182	0.021*	
H9B	0.678027	0.194711	0.721704	0.021*	
C10	0.6637 (5)	0.5215 (9)	0.76483 (19)	0.0160 (9)	
H10A	0.580027	0.590708	0.790675	0.019*	
H10B	0.660987	0.628991	0.731717	0.019*	
C11	0.8693 (6)	0.5098 (9)	0.7959 (2)	0.0165 (9)	
H11A	0.870743	0.406446	0.829691	0.020*	
H11B	0.951671	0.434785	0.770482	0.020*	
C12	0.9500 (6)	0.7609 (8)	0.8145 (2)	0.0172 (9)	
H12A	0.865418	0.837972	0.838936	0.021*	
H12B	0.952258	0.862509	0.780571	0.021*	
C13	1.1527 (6)	0.7484 (8)	0.8471 (2)	0.0170 (9)	
H13A	1.149900	0.648491	0.881296	0.020*	
H13B	1.236680	0.668910	0.822859	0.020*	
C14	1.2354 (6)	0.9980 (9)	0.8651 (2)	0.0183 (9)	
H14A	1.151432	1.077765	0.889286	0.022*	
H14B	1.238637	1.097924	0.830894	0.022*	
C15	1.4371 (6)	0.9839 (9)	0.8976 (2)	0.0191 (10)	
H15A	1.433404	0.884511	0.931853	0.023*	
H15B	1.520532	0.902806	0.873456	0.023*	
C16	1.5227 (6)	1.2328 (9)	0.9156 (2)	0.0207 (10)	
H16A	1.437411	1.316001	0.938848	0.025*	
H16B	1.530016	1.330526	0.881305	0.025*	
C17	1.7225 (6)	1.2177 (10)	0.9498 (2)	0.0231 (11)	
H17A	1.714132	1.126633	0.984997	0.028*	
H17B	1.806347	1.127749	0.927284	0.028*	
C18	1.8111 (7)	1.4663 (11)	0.9652 (2)	0.0277 (11)	
H18A	1.826747	1.554178	0.930507	0.042*	
H18B	1.933962	1.444704	0.987558	0.042*	
H18C	1.728567	1.557221	0.987212	0.042*	
H4	−0.207 (9)	−0.459 (13)	0.475 (2)	0.042*	

### 6-(Dodecyloxy)hexahydrofuro[3,2-b]furan-3-ol (iso-c12) Atomic displacement parameters (Å^2^)

**Table:**

	*U*^11^	*U*^22^	*U*^33^	*U*^12^	*U*^13^	*U*^23^
O4	0.0071 (12)	0.0232 (18)	0.0277 (16)	0.0025 (13)	0.0006 (11)	−0.0055 (14)
C5	0.0054 (16)	0.013 (2)	0.028 (2)	0.0004 (15)	0.0016 (15)	−0.0010 (17)
C6	0.0092 (17)	0.013 (2)	0.030 (2)	−0.0021 (17)	0.0029 (15)	−0.0012 (19)
O3	0.0127 (13)	0.0239 (18)	0.0265 (16)	−0.0038 (13)	0.0048 (11)	0.0004 (14)
C3	0.0072 (17)	0.010 (2)	0.031 (2)	0.0029 (16)	0.0021 (15)	−0.0011 (18)
C4	0.0070 (17)	0.0063 (18)	0.029 (2)	0.0008 (16)	0.0033 (15)	−0.0031 (17)
O1	0.0075 (12)	0.0128 (15)	0.0262 (16)	0.0019 (11)	0.0044 (11)	0.0004 (12)
C1	0.0082 (16)	0.017 (2)	0.026 (2)	0.0018 (17)	0.0013 (15)	0.0008 (19)
C2	0.0085 (16)	0.008 (2)	0.024 (2)	0.0031 (16)	0.0011 (14)	0.0010 (16)
O2	0.0072 (12)	0.0112 (15)	0.0304 (17)	−0.0017 (12)	−0.0016 (11)	−0.0012 (13)
C7	0.0082 (17)	0.016 (2)	0.031 (2)	0.0039 (18)	−0.0027 (15)	−0.002 (2)
C8	0.0109 (17)	0.012 (2)	0.027 (2)	−0.0018 (17)	0.0010 (15)	0.0008 (19)
C9	0.0116 (18)	0.015 (2)	0.025 (2)	0.0047 (17)	0.0006 (15)	−0.0034 (18)
C10	0.0088 (17)	0.018 (2)	0.021 (2)	−0.0001 (17)	0.0019 (14)	−0.0033 (18)
C11	0.0094 (17)	0.012 (2)	0.028 (2)	0.0035 (16)	0.0002 (15)	−0.0023 (18)
C12	0.0087 (17)	0.015 (2)	0.028 (2)	−0.0014 (17)	0.0022 (15)	−0.0015 (19)
C13	0.0082 (17)	0.013 (2)	0.029 (2)	0.0031 (16)	0.0003 (15)	−0.0027 (18)
C14	0.0102 (17)	0.016 (2)	0.028 (2)	0.0011 (17)	−0.0003 (15)	−0.0008 (19)
C15	0.0094 (18)	0.020 (2)	0.028 (2)	0.0013 (18)	0.0024 (16)	−0.0025 (19)
C16	0.0106 (18)	0.024 (3)	0.028 (2)	−0.0006 (18)	0.0017 (16)	0.002 (2)
C17	0.0114 (19)	0.027 (3)	0.031 (2)	−0.0001 (19)	0.0006 (17)	−0.001 (2)
C18	0.019 (2)	0.032 (3)	0.032 (3)	−0.005 (2)	−0.0002 (18)	−0.002 (2)

### 6-(Dodecyloxy)hexahydrofuro[3,2-b]furan-3-ol (iso-c12) Geometric parameters (Å, º)

**Table:**

O4—C5	1.414 (5)	C9—C10	1.529 (6)
O4—H4	0.90 (7)	C10—H10A	0.9700
C5—H5	0.9800	C10—H10B	0.9700
C5—C6	1.516 (6)	C10—C11	1.536 (5)
C5—C4	1.537 (6)	C11—H11A	0.9700
C6—H6A	0.9700	C11—H11B	0.9700
C6—H6B	0.9700	C11—C12	1.528 (6)
C6—O3	1.431 (5)	C12—H12A	0.9700
O3—C3	1.449 (5)	C12—H12B	0.9700
C3—H3	0.9800	C12—C13	1.532 (5)
C3—C4	1.514 (6)	C13—H13A	0.9700
C3—C2	1.538 (5)	C13—H13B	0.9700
C4—H4A	0.9800	C13—C14	1.522 (6)
C4—O1	1.447 (4)	C14—H14A	0.9700
O1—C1	1.457 (5)	C14—H14B	0.9700
C1—H1A	0.9700	C14—C15	1.526 (6)
C1—H1B	0.9700	C15—H15A	0.9700
C1—C2	1.519 (6)	C15—H15B	0.9700
C2—H2	0.9800	C15—C16	1.526 (7)
C2—O2	1.425 (5)	C16—H16A	0.9700
O2—C7	1.435 (5)	C16—H16B	0.9700
C7—H7A	0.9700	C16—C17	1.530 (6)
C7—H7B	0.9700	C17—H17A	0.9700
C7—C8	1.510 (6)	C17—H17B	0.9700
C8—H8A	0.9700	C17—C18	1.520 (7)
C8—H8B	0.9700	C18—H18A	0.9600
C8—C9	1.539 (5)	C18—H18B	0.9600
C9—H9A	0.9700	C18—H18C	0.9600
C9—H9B	0.9700		
			
C5—O4—H4	107 (4)	C10—C9—H9A	109.0
O4—C5—H5	107.8	C10—C9—H9B	109.0
O4—C5—C6	116.2 (4)	C9—C10—H10A	109.2
O4—C5—C4	115.2 (3)	C9—C10—H10B	109.2
C6—C5—H5	107.8	C9—C10—C11	112.3 (3)
C6—C5—C4	101.5 (3)	H10A—C10—H10B	107.9
C4—C5—H5	107.8	C11—C10—H10A	109.2
C5—C6—H6A	110.9	C11—C10—H10B	109.2
C5—C6—H6B	110.9	C10—C11—H11A	109.0
H6A—C6—H6B	108.9	C10—C11—H11B	109.0
O3—C6—C5	104.4 (4)	H11A—C11—H11B	107.8
O3—C6—H6A	110.9	C12—C11—C10	113.0 (3)
O3—C6—H6B	110.9	C12—C11—H11A	109.0
C6—O3—C3	108.6 (3)	C12—C11—H11B	109.0
O3—C3—H3	111.3	C11—C12—H12A	109.0
O3—C3—C4	107.1 (3)	C11—C12—H12B	109.0
O3—C3—C2	110.7 (3)	C11—C12—C13	113.1 (3)
C4—C3—H3	111.3	H12A—C12—H12B	107.8
C4—C3—C2	105.0 (3)	C13—C12—H12A	109.0
C2—C3—H3	111.3	C13—C12—H12B	109.0
C5—C4—H4A	111.7	C12—C13—H13A	108.9
C3—C4—C5	103.4 (3)	C12—C13—H13B	108.9
C3—C4—H4A	111.7	H13A—C13—H13B	107.7
O1—C4—C5	110.9 (3)	C14—C13—C12	113.4 (3)
O1—C4—C3	107.0 (3)	C14—C13—H13A	108.9
O1—C4—H4A	111.7	C14—C13—H13B	108.9
C4—O1—C1	109.2 (3)	C13—C14—H14A	109.0
O1—C1—H1A	110.7	C13—C14—H14B	109.0
O1—C1—H1B	110.7	C13—C14—C15	113.1 (4)
O1—C1—C2	105.4 (3)	H14A—C14—H14B	107.8
H1A—C1—H1B	108.8	C15—C14—H14A	109.0
C2—C1—H1A	110.7	C15—C14—H14B	109.0
C2—C1—H1B	110.7	C14—C15—H15A	108.8
C3—C2—H2	111.4	C14—C15—H15B	108.8
C1—C2—C3	101.9 (3)	H15A—C15—H15B	107.7
C1—C2—H2	111.4	C16—C15—C14	113.7 (4)
O2—C2—C3	106.2 (3)	C16—C15—H15A	108.8
O2—C2—C1	114.0 (3)	C16—C15—H15B	108.8
O2—C2—H2	111.4	C15—C16—H16A	108.8
C2—O2—C7	112.7 (3)	C15—C16—H16B	108.8
O2—C7—H7A	109.9	C15—C16—C17	113.6 (4)
O2—C7—H7B	109.9	H16A—C16—H16B	107.7
O2—C7—C8	109.2 (3)	C17—C16—H16A	108.8
H7A—C7—H7B	108.3	C17—C16—H16B	108.8
C8—C7—H7A	109.9	C16—C17—H17A	108.9
C8—C7—H7B	109.9	C16—C17—H17B	108.9
C7—C8—H8A	109.4	H17A—C17—H17B	107.7
C7—C8—H8B	109.4	C18—C17—C16	113.5 (4)
C7—C8—C9	111.2 (3)	C18—C17—H17A	108.9
H8A—C8—H8B	108.0	C18—C17—H17B	108.9
C9—C8—H8A	109.4	C17—C18—H18A	109.5
C9—C8—H8B	109.4	C17—C18—H18B	109.5
C8—C9—H9A	109.0	C17—C18—H18C	109.5
C8—C9—H9B	109.0	H18A—C18—H18B	109.5
H9A—C9—H9B	107.8	H18A—C18—H18C	109.5
C10—C9—C8	112.9 (3)	H18B—C18—H18C	109.5
			
O4—C5—C6—O3	164.6 (3)	C4—O1—C1—C2	−25.6 (4)
O4—C5—C4—C3	−158.5 (3)	O1—C1—C2—C3	33.7 (4)
O4—C5—C4—O1	−44.1 (5)	O1—C1—C2—O2	−80.2 (4)
C5—C6—O3—C3	−31.0 (4)	C1—C2—O2—C7	−77.0 (4)
C5—C4—O1—C1	−105.9 (4)	C2—C3—C4—C5	132.5 (3)
C6—C5—C4—C3	−32.1 (4)	C2—C3—C4—O1	15.4 (4)
C6—C5—C4—O1	82.3 (4)	C2—O2—C7—C8	−172.5 (3)
C6—O3—C3—C4	10.0 (4)	O2—C7—C8—C9	179.4 (3)
C6—O3—C3—C2	−103.9 (4)	C7—C8—C9—C10	−179.1 (4)
O3—C3—C4—C5	14.7 (4)	C8—C9—C10—C11	−175.0 (3)
O3—C3—C4—O1	−102.4 (3)	C9—C10—C11—C12	178.0 (4)
O3—C3—C2—C1	85.6 (4)	C10—C11—C12—C13	178.2 (3)
O3—C3—C2—O2	−154.8 (3)	C11—C12—C13—C14	179.2 (4)
C3—C4—O1—C1	6.2 (4)	C12—C13—C14—C15	179.9 (4)
C3—C2—O2—C7	171.6 (3)	C13—C14—C15—C16	179.6 (4)
C4—C5—C6—O3	38.9 (4)	C14—C15—C16—C17	178.3 (4)
C4—C3—C2—C1	−29.7 (4)	C15—C16—C17—C18	177.3 (4)
C4—C3—C2—O2	89.9 (4)		

### 6-(Tetradecyloxy)hexahydrofuro[3,2-b]furan-3-ol (iso-c14) Crystal data

**Table:**

C_20_H_38_O_4_	*F*(000) = 380
*M**_r_* = 342.50	*D*_x_ = 1.163 Mg m^−^^3^
Monoclinic, *P*2_1_	Mo *K*α radiation, λ = 0.71073 Å
*a* = 7.040 (6) Å	Cell parameters from 1866 reflections
*b* = 5.438 (5) Å	θ = 2.4–23.7°
*c* = 25.56 (2) Å	µ = 0.08 mm^−^^1^
β = 91.914 (9)°	*T* = 100 K
*V* = 978.2 (14) Å^3^	Plate, colourless
*Z* = 2	0.3 × 0.1 × 0.02 mm

### 6-(Tetradecyloxy)hexahydrofuro[3,2-b]furan-3-ol (iso-c14) Data collection

**Table:**

Bruker APEXII CCD diffractometer	2949 reflections with *I* > 2σ(*I*)
Detector resolution: 8.3 pixels mm^-1^	*R*_int_ = 0.069
φ and ω scans	θ_max_ = 27.1°, θ_min_ = 2.4°
Absorption correction: numerical (SADABS; Bruker, 2016)	*h* = −9→9
*T*_min_ = 0.543, *T*_max_ = 0.746	*k* = −6→6
12041 measured reflections	*l* = −32→32
4278 independent reflections	

### 6-(Tetradecyloxy)hexahydrofuro[3,2-b]furan-3-ol (iso-c14) Refinement

**Table:**

Refinement on *F*^2^	Hydrogen site location: mixed
Least-squares matrix: full	H atoms treated by a mixture of independent and constrained refinement
*R*[*F*^2^ > 2σ(*F*^2^)] = 0.055	*w* = 1/[σ^2^(*F*_o_^2^) + (0.0562*P*)^2^] where *P* = (*F*_o_^2^ + 2*F*_c_^2^)/3
*wR*(*F*^2^) = 0.140	(Δ/σ)_max_ < 0.001
*S* = 1.04	Δρ_max_ = 0.22 e Å^−^^3^
4278 reflections	Δρ_min_ = −0.23 e Å^−^^3^
221 parameters	Absolute structure: Flack *x* determined using 980 quotients [(*I*^+^)-(*I*^-^)]/[(*I*^+^)+(*I*^-^)] (Parsons *et al.*, 2013)
1 restraint	Absolute structure parameter: −1.2 (10)
Primary atom site location: dual	

### 6-(Tetradecyloxy)hexahydrofuro[3,2-b]furan-3-ol (iso-c14) Special details

**Table:**

Geometry. All esds (except the esd in the dihedral angle between two l.s. planes) are estimated using the full covariance matrix. The cell esds are taken into account individually in the estimation of esds in distances, angles and torsion angles; correlations between esds in cell parameters are only used when they are defined by crystal symmetry. An approximate (isotropic) treatment of cell esds is used for estimating esds involving l.s. planes.

### 6-(Tetradecyloxy)hexahydrofuro[3,2-b]furan-3-ol (iso-c14) Fractional atomic coordinates and isotropic or equivalent isotropic displacement parameters (Å^2^)

**Table:**

	*x*	*y*	*z*	*U*_iso_*/*U*_eq_	
O4	−0.2928 (3)	−0.4743 (5)	0.52486 (10)	0.0275 (6)	
H4	−0.194 (6)	−0.559 (8)	0.5245 (16)	0.041*	
O3	−0.3534 (3)	−0.4511 (5)	0.38374 (9)	0.0296 (6)	
C6	−0.3661 (5)	−0.5840 (7)	0.43214 (14)	0.0264 (8)	
H6A	−0.266785	−0.712630	0.435169	0.032*	
H6B	−0.492403	−0.661957	0.434930	0.032*	
C5	−0.3353 (5)	−0.3874 (7)	0.47378 (13)	0.0249 (8)	
H5	−0.453999	−0.286998	0.474978	0.030*	
C4	−0.1807 (5)	−0.2270 (7)	0.45005 (13)	0.0233 (8)	
H4A	−0.186530	−0.052784	0.462419	0.028*	
O1	0.0060 (3)	−0.3353 (5)	0.45978 (9)	0.0251 (6)	
C1	0.0720 (5)	−0.4407 (7)	0.41133 (13)	0.0261 (8)	
H1A	0.042202	−0.618421	0.409508	0.031*	
H1B	0.211101	−0.419197	0.408917	0.031*	
C2	−0.0320 (5)	−0.3032 (7)	0.36763 (14)	0.0247 (9)	
H2	−0.047190	−0.406857	0.335457	0.030*	
C3	−0.2239 (5)	−0.2490 (7)	0.39142 (14)	0.0246 (8)	
H3	−0.279669	−0.092979	0.377018	0.029*	
O2	0.0514 (3)	−0.0703 (5)	0.35619 (10)	0.0267 (6)	
C7	0.2197 (5)	−0.0927 (8)	0.32631 (15)	0.0286 (9)	
H7A	0.192742	−0.195626	0.294985	0.034*	
H7B	0.321577	−0.173310	0.347733	0.034*	
C8	0.2838 (5)	0.1587 (7)	0.30984 (15)	0.0264 (8)	
H8A	0.310314	0.260127	0.341405	0.032*	
H8B	0.180094	0.239026	0.289060	0.032*	
C9	0.4616 (5)	0.1482 (7)	0.27738 (15)	0.0274 (8)	
H9A	0.565128	0.069171	0.298453	0.033*	
H9B	0.435108	0.043818	0.246248	0.033*	
C10	0.5289 (5)	0.3998 (7)	0.25933 (14)	0.0254 (8)	
H10A	0.543981	0.509412	0.290107	0.031*	
H10B	0.430641	0.472216	0.235393	0.031*	
C11	0.7171 (5)	0.3883 (7)	0.23132 (15)	0.0274 (8)	
H11A	0.813774	0.309314	0.254778	0.033*	
H11B	0.700338	0.283559	0.199816	0.033*	
C12	0.7903 (5)	0.6391 (8)	0.21489 (15)	0.0269 (8)	
H12A	0.810126	0.742408	0.246494	0.032*	
H12B	0.692301	0.719783	0.192145	0.032*	
C13	0.9754 (5)	0.6271 (7)	0.18580 (15)	0.0285 (9)	
H13A	0.955627	0.523019	0.154311	0.034*	
H13B	1.073413	0.546723	0.208622	0.034*	
C14	1.0495 (5)	0.8774 (7)	0.16902 (15)	0.0277 (9)	
H14A	0.949790	0.960044	0.147198	0.033*	
H14B	1.073387	0.979425	0.200626	0.033*	
C15	1.2308 (5)	0.8655 (7)	0.13841 (15)	0.0277 (9)	
H15A	1.206746	0.763969	0.106733	0.033*	
H15B	1.330330	0.782022	0.160177	0.033*	
C16	1.3060 (5)	1.1164 (7)	0.12173 (15)	0.0275 (9)	
H16A	1.208134	1.197719	0.099038	0.033*	
H16B	1.326405	1.219760	0.153298	0.033*	
C17	1.4905 (5)	1.1042 (7)	0.09257 (14)	0.0265 (9)	
H17A	1.469484	1.002373	0.060793	0.032*	
H17B	1.587719	1.020765	0.115103	0.032*	
C18	1.5675 (5)	1.3533 (7)	0.07642 (15)	0.0280 (9)	
H18A	1.592548	1.453521	0.108237	0.034*	
H18B	1.469066	1.439061	0.054693	0.034*	
C19	1.7495 (5)	1.3374 (7)	0.04580 (15)	0.0296 (9)	
H19A	1.846150	1.245600	0.066956	0.036*	
H19B	1.722972	1.242708	0.013287	0.036*	
C20	1.8309 (5)	1.5850 (8)	0.03147 (15)	0.0339 (10)	
H20A	1.736940	1.676491	0.010062	0.051*	
H20B	1.946100	1.560924	0.011619	0.051*	
H20C	1.862245	1.677821	0.063460	0.051*	

### 6-(Tetradecyloxy)hexahydrofuro[3,2-b]furan-3-ol (iso-c14) Atomic displacement parameters (Å^2^)

**Table:**

	*U*^11^	*U*^22^	*U*^33^	*U*^12^	*U*^13^	*U*^23^
O4	0.0230 (12)	0.0320 (16)	0.0280 (14)	0.0053 (12)	0.0071 (11)	0.0039 (12)
O3	0.0286 (13)	0.0301 (15)	0.0303 (14)	−0.0067 (12)	0.0027 (11)	0.0013 (12)
C6	0.0262 (18)	0.024 (2)	0.030 (2)	−0.0015 (16)	0.0044 (15)	0.0053 (17)
C5	0.0200 (17)	0.027 (2)	0.028 (2)	0.0023 (15)	0.0039 (14)	0.0032 (17)
C4	0.0237 (18)	0.0194 (18)	0.027 (2)	0.0050 (16)	0.0022 (14)	0.0019 (16)
O1	0.0198 (12)	0.0271 (15)	0.0288 (14)	0.0010 (11)	0.0045 (10)	0.0001 (11)
C1	0.0271 (18)	0.025 (2)	0.0265 (19)	−0.0015 (16)	0.0041 (15)	−0.0032 (17)
C2	0.0250 (18)	0.022 (2)	0.027 (2)	−0.0034 (16)	0.0041 (15)	0.0006 (16)
C3	0.0255 (19)	0.0197 (19)	0.029 (2)	0.0009 (16)	0.0031 (15)	0.0018 (16)
O2	0.0261 (13)	0.0242 (15)	0.0305 (15)	−0.0023 (11)	0.0102 (11)	0.0011 (12)
C7	0.0239 (18)	0.031 (2)	0.031 (2)	−0.0005 (17)	0.0083 (15)	0.0042 (18)
C8	0.0256 (18)	0.0230 (19)	0.031 (2)	0.0007 (16)	0.0059 (15)	0.0022 (17)
C9	0.0267 (19)	0.027 (2)	0.028 (2)	0.0012 (17)	0.0065 (15)	0.0039 (17)
C10	0.0237 (18)	0.028 (2)	0.025 (2)	−0.0006 (16)	0.0043 (14)	0.0010 (17)
C11	0.0253 (18)	0.028 (2)	0.029 (2)	0.0004 (17)	0.0066 (15)	0.0036 (17)
C12	0.0237 (18)	0.031 (2)	0.027 (2)	0.0004 (16)	0.0051 (15)	0.0013 (17)
C13	0.0236 (18)	0.033 (2)	0.029 (2)	0.0011 (17)	0.0054 (15)	0.0014 (18)
C14	0.0254 (18)	0.028 (2)	0.030 (2)	−0.0018 (17)	0.0048 (15)	−0.0018 (17)
C15	0.0258 (19)	0.026 (2)	0.032 (2)	0.0011 (16)	0.0040 (15)	0.0016 (17)
C16	0.0242 (19)	0.029 (2)	0.030 (2)	−0.0021 (16)	0.0051 (15)	0.0010 (17)
C17	0.0272 (18)	0.023 (2)	0.030 (2)	0.0006 (16)	0.0046 (15)	0.0037 (17)
C18	0.0261 (19)	0.027 (2)	0.031 (2)	−0.0012 (16)	0.0065 (16)	0.0003 (17)
C19	0.026 (2)	0.029 (2)	0.033 (2)	0.0006 (17)	0.0029 (16)	0.0006 (17)
C20	0.030 (2)	0.033 (3)	0.039 (2)	−0.0057 (18)	0.0091 (17)	0.0007 (19)

### 6-(Tetradecyloxy)hexahydrofuro[3,2-b]furan-3-ol (iso-c14) Geometric parameters (Å, º)

**Table:**

O4—H4	0.83 (4)	C10—C11	1.528 (5)
O4—C5	1.411 (4)	C11—H11A	0.9900
O3—C6	1.438 (4)	C11—H11B	0.9900
O3—C3	1.438 (4)	C11—C12	1.522 (5)
C6—H6A	0.9900	C12—H12A	0.9900
C6—H6B	0.9900	C12—H12B	0.9900
C6—C5	1.519 (5)	C12—C13	1.523 (5)
C5—H5	1.0000	C13—H13A	0.9900
C5—C4	1.535 (5)	C13—H13B	0.9900
C4—H4A	1.0000	C13—C14	1.524 (5)
C4—O1	1.454 (4)	C14—H14A	0.9900
C4—C3	1.524 (5)	C14—H14B	0.9900
O1—C1	1.455 (4)	C14—C15	1.521 (5)
C1—H1A	0.9900	C15—H15A	0.9900
C1—H1B	0.9900	C15—H15B	0.9900
C1—C2	1.513 (5)	C15—C16	1.529 (5)
C2—H2	1.0000	C16—H16A	0.9900
C2—C3	1.528 (5)	C16—H16B	0.9900
C2—O2	1.430 (4)	C16—C17	1.520 (5)
C3—H3	1.0000	C17—H17A	0.9900
O2—C7	1.437 (4)	C17—H17B	0.9900
C7—H7A	0.9900	C17—C18	1.521 (5)
C7—H7B	0.9900	C18—H18A	0.9900
C7—C8	1.504 (5)	C18—H18B	0.9900
C8—H8A	0.9900	C18—C19	1.526 (5)
C8—H8B	0.9900	C19—H19A	0.9900
C8—C9	1.526 (5)	C19—H19B	0.9900
C9—H9A	0.9900	C19—C20	1.513 (6)
C9—H9B	0.9900	C20—H20A	0.9800
C9—C10	1.525 (5)	C20—H20B	0.9800
C10—H10A	0.9900	C20—H20C	0.9800
C10—H10B	0.9900		
			
C5—O4—H4	109 (3)	C11—C10—H10A	109.0
C3—O3—C6	108.9 (3)	C11—C10—H10B	109.0
O3—C6—H6A	111.0	C10—C11—H11A	108.9
O3—C6—H6B	111.0	C10—C11—H11B	108.9
O3—C6—C5	103.7 (3)	H11A—C11—H11B	107.7
H6A—C6—H6B	109.0	C12—C11—C10	113.5 (3)
C5—C6—H6A	111.0	C12—C11—H11A	108.9
C5—C6—H6B	111.0	C12—C11—H11B	108.9
O4—C5—C6	115.7 (3)	C11—C12—H12A	108.9
O4—C5—H5	107.8	C11—C12—H12B	108.9
O4—C5—C4	115.1 (3)	C11—C12—C13	113.5 (3)
C6—C5—H5	107.8	H12A—C12—H12B	107.7
C6—C5—C4	102.1 (3)	C13—C12—H12A	108.9
C4—C5—H5	107.8	C13—C12—H12B	108.9
C5—C4—H4A	112.1	C12—C13—H13A	108.8
O1—C4—C5	110.5 (3)	C12—C13—H13B	108.8
O1—C4—H4A	112.1	C12—C13—C14	113.9 (3)
O1—C4—C3	106.6 (3)	H13A—C13—H13B	107.7
C3—C4—C5	102.9 (3)	C14—C13—H13A	108.8
C3—C4—H4A	112.1	C14—C13—H13B	108.8
C4—O1—C1	109.1 (3)	C13—C14—H14A	108.7
O1—C1—H1A	110.6	C13—C14—H14B	108.7
O1—C1—H1B	110.6	H14A—C14—H14B	107.6
O1—C1—C2	105.9 (3)	C15—C14—C13	114.1 (3)
H1A—C1—H1B	108.7	C15—C14—H14A	108.7
C2—C1—H1A	110.6	C15—C14—H14B	108.7
C2—C1—H1B	110.6	C14—C15—H15A	108.7
C1—C2—H2	111.4	C14—C15—H15B	108.7
C1—C2—C3	102.4 (3)	C14—C15—C16	114.2 (3)
C3—C2—H2	111.4	H15A—C15—H15B	107.6
O2—C2—C1	113.4 (3)	C16—C15—H15A	108.7
O2—C2—H2	111.4	C16—C15—H15B	108.7
O2—C2—C3	106.6 (3)	C15—C16—H16A	108.7
O3—C3—C4	107.4 (3)	C15—C16—H16B	108.7
O3—C3—C2	111.3 (3)	H16A—C16—H16B	107.6
O3—C3—H3	111.0	C17—C16—C15	114.0 (3)
C4—C3—C2	104.9 (3)	C17—C16—H16A	108.7
C4—C3—H3	111.0	C17—C16—H16B	108.7
C2—C3—H3	111.0	C16—C17—H17A	108.7
C2—O2—C7	112.7 (3)	C16—C17—H17B	108.7
O2—C7—H7A	109.8	C16—C17—C18	114.3 (3)
O2—C7—H7B	109.8	H17A—C17—H17B	107.6
O2—C7—C8	109.4 (3)	C18—C17—H17A	108.7
H7A—C7—H7B	108.2	C18—C17—H17B	108.7
C8—C7—H7A	109.8	C17—C18—H18A	108.8
C8—C7—H7B	109.8	C17—C18—H18B	108.8
C7—C8—H8A	109.2	C17—C18—C19	113.7 (3)
C7—C8—H8B	109.2	H18A—C18—H18B	107.7
C7—C8—C9	112.2 (3)	C19—C18—H18A	108.8
H8A—C8—H8B	107.9	C19—C18—H18B	108.8
C9—C8—H8A	109.2	C18—C19—H19A	108.8
C9—C8—H8B	109.2	C18—C19—H19B	108.8
C8—C9—H9A	108.8	H19A—C19—H19B	107.7
C8—C9—H9B	108.8	C20—C19—C18	113.9 (3)
H9A—C9—H9B	107.7	C20—C19—H19A	108.8
C10—C9—C8	113.6 (3)	C20—C19—H19B	108.8
C10—C9—H9A	108.8	C19—C20—H20A	109.5
C10—C9—H9B	108.8	C19—C20—H20B	109.5
C9—C10—H10A	109.0	C19—C20—H20C	109.5
C9—C10—H10B	109.0	H20A—C20—H20B	109.5
C9—C10—C11	112.8 (3)	H20A—C20—H20C	109.5
H10A—C10—H10B	107.8	H20B—C20—H20C	109.5
			
O4—C5—C4—O1	44.5 (4)	C2—O2—C7—C8	172.6 (3)
O4—C5—C4—C3	158.0 (3)	C3—O3—C6—C5	31.2 (3)
O3—C6—C5—O4	−164.6 (3)	C3—C4—O1—C1	−4.8 (4)
O3—C6—C5—C4	−38.8 (3)	C3—C2—O2—C7	−171.1 (3)
C6—O3—C3—C4	−10.6 (3)	O2—C2—C3—O3	154.9 (3)
C6—O3—C3—C2	103.7 (3)	O2—C2—C3—C4	−89.3 (3)
C6—C5—C4—O1	−81.7 (3)	O2—C7—C8—C9	−179.4 (3)
C6—C5—C4—C3	31.8 (3)	C7—C8—C9—C10	179.2 (3)
C5—C4—O1—C1	106.3 (3)	C8—C9—C10—C11	174.6 (3)
C5—C4—C3—O3	−14.1 (3)	C9—C10—C11—C12	−177.8 (3)
C5—C4—C3—C2	−132.6 (3)	C10—C11—C12—C13	−178.6 (3)
C4—O1—C1—C2	24.4 (4)	C11—C12—C13—C14	179.8 (3)
O1—C4—C3—O3	102.2 (3)	C12—C13—C14—C15	−178.1 (3)
O1—C4—C3—C2	−16.3 (4)	C13—C14—C15—C16	−179.7 (3)
O1—C1—C2—C3	−33.4 (3)	C14—C15—C16—C17	178.3 (3)
O1—C1—C2—O2	81.0 (3)	C15—C16—C17—C18	−179.3 (3)
C1—C2—C3—O3	−85.9 (3)	C16—C17—C18—C19	−178.3 (3)
C1—C2—C3—C4	30.0 (4)	C17—C18—C19—C20	−177.7 (3)
C1—C2—O2—C7	77.0 (4)		

## References

[bb1] Bruker (2016). *BlS* and *SADABS*. Bruker AXS Inc., Madison, Wisconsin, USA.

[bb2] Bruker (2019). *SAINT*. Bruker AXS Inc., Madison, Wisconsin, USA.

[bb3] Dolomanov, O. V., Bourhis, L. J., Gildea, R. J., Howard, J. A. K. & Puschmann, H. (2009). *J. Appl. Cryst.* **42**, 339–341.

[bb4] Etzbach, K.-H., Delavier, P., Siemensmeyer, K., Wagenblast, G., Laupichler, L. & Vill, V. (1995). DE Patent 4342280.

[bb5] Flack, H. D. (2008). *Acta Chim. Slov.* **55**, 689–691.

[bb6] Flack, H. D. & Bernardinelli, G. (2000). *J. Appl. Cryst.* **33**, 1143–1148.

[bb7] Flack, H. D. & Shmueli, U. (2007). *Acta Cryst.* A**63**, 257–265. 10.1107/S0108767307002802 17435290

[bb8] Groom, C. R., Bruno, I. J., Lightfoot, M. P. & Ward, S. C. (2016). *Acta Cryst.* B**72**, 171–179. 10.1107/S2052520616003954 PMC4822653 27048719

[bb19] Harata, K. & Kawano, K. (2002). *Carbohydr. Res.* **337**, 537–547. 10.1016/s0008-6215(02)00019-8 11890891

[bb20] Hušák, M., Kratochvíl, B., Jegorov, A., Mat’ha, V., Stuchlík, M. & Andrýsek, T. (1996). *Z. Kristallogr.* **211**, 313–318.

[bb9] Kanters, J. A., Schouten, A., Sterk, G. J. & de Jong, M. H. (1993). *J. Mol. Struct.* **298**, 113–120.

[bb10] Parsons, S., Flack, H. D. & Wagner, T. (2013). *Acta Cryst.* B**69**, 249–259. 10.1107/S2052519213010014 PMC3661305 23719469

[bb11] Rigaku OD (2020). *CrysAlis PRO*. Rigaku Oxford Diffraction, Yarnton, England.

[bb12] Sagawa, T., Kobayashi, H., Murata, C., Shichibu, Y., Konishi, K. & Fukuoka, A. (2019). *ACS Sustainable Chem. Eng.* **7**, 14883-14888.

[bb13] Santschi, N., Wagner, S., Daniliuc, C., Hermann, S., Schäfers, M. & Gilmour, R. (2015). *ChemMedChem*, **10**, 1724–1732. 10.1002/cmdc.201500275 26267858

[bb14] Sheldrick, G. M. (2015*a*). *Acta Cryst.* A**71**, 3–8.

[bb15] Sheldrick, G. M. (2015*b*). *Acta Cryst.* C**71**, 3–8.

[bb16] Vill, V., Fischer, F. & Thiem, J. (1988). *Z. Naturforsch. Teil A*, **42**, 1119–1125.

[bb17] Vill, V., Fischer, F. & Thiem, J. (1989). *Z. Naturforsch. Teil A*, **43**, 675–679.

[bb21] Wu, J., Thiyagarajan, S., Guerra, C. F., Eduard, P., Lutz, M., Noordover, B. A. J., Koning, C. E., van Es, D. S. (2017). *ChemSusChem*, **10**, 3202–3211. 10.1002/cssc.201700617 28590079

[bb18] Zhu, Y., Durand, M., Molinier, V. & Aubry, J.-M. (2008). *Green Chem.* **10**, 532–540.

